# Well-positioned nucleosomes punctuate polycistronic pol II transcription units and flank silent *VSG* gene arrays in *Trypanosoma brucei*

**DOI:** 10.1186/s13072-017-0121-9

**Published:** 2017-03-20

**Authors:** Johannes Petrus Maree, Megan Lindsay Povelones, David Johannes Clark, Gloria Rudenko, Hugh-George Patterton

**Affiliations:** 1 Department of Biochemistry, Stellenbosch University, Matieland, 7602 South Africa; 2 Department of Biology, Pennsylvania State University (Brandywine Campus), Media, PA 19063 USA; 3 Division of Developmental Biology, Eunice Kennedy Shriver National Institute for Child Health and Human Development, National Institutes of Health, Bethesda, MD USA; 4 Department of Life Sciences, Imperial College London, South Kensington, London, SW7 2AZ UK

**Keywords:** Genome-wide nucleosome positions, *Trypanosoma brucei*, Polycistronic transcription units, Silent variant surface glycoproteins, *VSG*, Bloodstream expression sites, Evolution, MNase-seq

## Abstract

**Background:**

The compaction of DNA in chromatin in eukaryotes allowed the expansion of genome size and coincided with significant evolutionary diversification. However, chromatin generally represses DNA function, and mechanisms coevolved to regulate chromatin structure and its impact on DNA. This included the selection of specific nucleosome positions to modulate accessibility to the DNA molecule. *Trypanosoma brucei*, a member of the Excavates supergroup, falls in an ancient evolutionary branch of eukaryotes and provides valuable insight into the organization of chromatin in early genomes.

**Results:**

We have mapped nucleosome positions in *T. brucei* and identified important differences compared to other eukaryotes: The RNA polymerase II initiation regions in *T. brucei* do not exhibit pronounced nucleosome depletion, and show little evidence for defined −1 and +1 nucleosomes. In contrast, a well-positioned nucleosome is present directly on the splice acceptor sites within the polycistronic transcription units. The RNA polyadenylation sites were depleted of nucleosomes, with a single well-positioned nucleosome present immediately downstream of the predicted sites. The regions flanking the silent variant surface glycoprotein (VSG) gene cassettes showed extensive arrays of well-positioned nucleosomes, which may repress cryptic transcription initiation. The silent VSG genes themselves exhibited a less regular nucleosomal pattern in both bloodstream and procyclic form trypanosomes. The DNA replication origins, when present within silent VSG gene cassettes, displayed a defined nucleosomal organization compared with replication origins in other chromosomal core regions.

**Conclusions:**

Our results indicate that some organizational features of chromatin are evolutionarily ancient, and may already have been present in the last eukaryotic common ancestor.

**Electronic supplementary material:**

The online version of this article (doi:10.1186/s13072-017-0121-9) contains supplementary material, which is available to authorized users.

## Background

African trypanosomes lie in an ancient evolutionary branch of the Excavates supergroup, which split from the last eukaryotic common ancestor (LECA) along with the SAR, Archaeplastida, and Unikonta supergroups some 2 billion years ago [1, 2]. Despite this early divergence, *Trypanosoma brucei* encodes an extensive repertoire of proteins associated with chromatin structure, modification, and functional regulation [3–6]. The presence of an epigenome in trypanosomes is perhaps expected, given the evolutionary origin of functional core histones in the ancestral Archaea [7], and the presence of linker histone homologs in evolutionarily distant bacteria [8]. Although Archaea lack multi-domain chromatin remodelers [9], the SNF2 domain, which has DNA-dependent ATPase activity and is present in a broad range of chromatin remodelers [10], is identifiable in bacterial helicases. The occurrence of histone modification enzymes, and a functional role for modified DNA packaging proteins, was also demonstrated in Archaea [11], suggesting that the regulation of chromatin structure and the epigenetic definition of different functional states of chromatin predates LECA.

Nucleosomes can influence diverse DNA functions [12–14], and the precise nucleosome positions present in regulatory elements are functionally crucial [15]. Nucleosomes can also assume a similarly defined distribution around RNA polymerase II (pol II) transcription start sites (TSSs) in diverse eukaryotes from the Unikonta supergroup, including *Dictyostelium discoideum*, *Saccharomyces cerevisiae*, and *Homo sapiens*. Incredibly, this nucleosomal arrangement appears evolutionarily ancient, as a similar nucleosome-depleted region bracketed by positioned −1 and +1 “nucleosomes” was observed upstream of genes in the archaeal *Haloferax volcanii*. This is despite the fact that the structural chromatin unit in this archaeal cell is composed of a tetramer instead of an octamer of histones [16].

There is currently a lack of insight into the genome-wide nucleosomal organization of genomes from organisms that are evolutionarily far removed from the Unikonta supergroup of eukaryotes, yet encode conventional nucleosomes composed of a conserved octamer of histones. In this regard, *T. brucei* represents a very intriguing subject. Here, all four core histones are present, and the classic conservation benchmark, canonical H4, is 79% similar to that of *H. sapiens*. The most recent ancestor shared between *T. brucei* and the Unikonta supergroup is therefore, in all likelihood, LECA.

*Trypanosoma brucei* is a unicellular parasite that is transmitted to humans by one of several *Glossina* fly species, and causes human African trypanosomiasis (HAT) [17]. Upon initial human infection, *T. brucei* invades interstitial spaces, the lymph system, and the bloodstream. With prolonged infection, the parasite crosses the blood–brain barrier and invades the central nervous system [18]. Without treatment HAT is often fatal and although the number of cases is declining, more than 1.8 million people are still thought to be at high risk of the disease [19]. As the parasite cycles between the mammalian host and the insect vector, it differentiates into different life cycle stages including the bloodstream form (BF) in the mammal or the procyclic form (PF) in the midgut of the tsetse fly [20].

In the bloodstream of the mammalian host, *T. brucei* escapes clearance by the immune system by periodically switching a mono-allelically expressed variant surface glycoprotein (*VSG*), an abundant cell surface protein that masks invariant cell surface proteins [21, 22]. The active *VSG* is expressed from a single pol I-transcribed subtelomeric *VSG* expression site (ES) [23]. The expressed *VSG* gene can be switched through multiple mechanisms [24]. First of all, a transcriptional switch can result in silencing of the active ES and the activation of one of approximately 15 other silent ESs. Alternatively, DNA recombination can be involved. Gene conversion can result in all or part of the active *VSG* gene being swapped with sequences from a different silent *VSG* cassette, present on a variety of types of chromosomes. *T. brucei* contains 11 megabase chromosomes (>1 Mb), ~5 intermediate chromosomes (200–900 kb), and ~100 mini-chromosomes (30–150 kb), and all of these contain silent *VSGs* [25]. Lastly, *VSG*s can be switched through telomere exchange with another *VSG*-containing telomere.

ESs are telomeric transcription units. There is a relatively localized telomeric silencing gradient extending up to 10 kb from the telomere end, although this is not implicated in the ES regulation involved in antigenic variation [26–29]. The telomeric repression observed in the immediate vicinity of the telomeres of the silent ESs appears superficially reminiscent of that observed in yeast and *Drosophila* in that it requires RAP1, among other factors [26]. However, SIR2, which plays an important role in telomere position effect in eukaryotes, appears to also have unrelated functions in *T. brucei* [29]. Additional repressive mechanisms appear to operate on the ES promoter itself. These is about 40–60-kb upstream from the chromosome end and is effectively silenced, even though distance-wise it would be expected to escape the effects of typical telomere position effect [30]. A number of proteins including the chromatin remodelers ISWI [31, 32], ORC1 [33], FACT [34], and HDAC3 [35] among others, play a role in ES promoter silencing. In addition, *T. brucei* histone H1, similar to the C-terminal tail of the H1 from metazoans, renders chromatin in the BF stage more resistant to nucleases, presumably due to a more closed chromatin conformation. H1 is also required for full transcriptional repression of silent ESs [36, 37]. Another unusual feature of ESs is that they are transcribed in a mono-allelic fashion by pol I, which in eukaryotes normally exclusively transcribes ribosomal DNA (rRNA) [38].

Unusually for a eukaryote, protein-coding genes in *T. brucei* are arranged in extensive, polycistronic transcription units (PTUs). These are constitutively transcribed by pol II from poorly defined promoters and can span up to several hundred kilobases [39]. There is no transcriptional regulation of pol II in *T. brucei*, and virtually all regulation of mRNA and protein levels appears to occur posttranscriptionally [40]. Pol II transcription initiation typically occurs in the strand switch regions (SSRs) between two divergently transcribed PTUs, but pol II start sites have also been identified between head-to-tail aligned PTUs [41]. Various epigenetic marks are located at SSRs and are likely to functionally define pol II regulatory regions [42]. The pol II transcription start sites are enriched for the H2A.V and H2B.V histone variants, as well as for modified H3K4me3 and H4K10ac [42, 43]. Termination of pol II transcription also occurs in the SSRs, for example, between two convergent PTUs. These termination regions are enriched for the H3.V and H4.V histone variants and modified H3K76me1/2 [42, 44]. In addition, the modified thymidine base, β-d-glucosyl-hydroxymethyluracil (base J), is also located at termination sites. Base J was shown to contribute to transcriptional termination in both *T. brucei* [45, 46] and *Leishmania tarentolae* [47], and the knockdown of base J and H3.V in *T. brucei* results in transcriptional read-through, and the appearance of downstream anti-sense RNA [45]. In addition, pol II termination is often also associated with a tRNA gene [42].

In this study, we mapped the genome-wide nucleosome positions in two different life cycle stages (BF and PF) of *T. brucei* 427 using MNase-seq. We report the nucleosomal organization at pol I and pol III promoters, as well as in regions flanking pol II-transcribed PTUs, including the adjacent transcription start and termination regions. We find that the pol II PTUs are punctuated internally by well-positioned nucleosomes at regions involved in RNA processing and analyzed the possible contribution of DNA sequence elements in the nucleosomal positions that were observed. In addition, we find that the silent *VSG* gene arrays are flanked by regions of well-positioned nucleosomes. This could play a role in suppressing fortuitous initiation by pol II, and ensuring mono-allelic expression of a single *VSG* from the active ES.

## Results

### Distribution of positioned nucleosomes in the *T. brucei* genome

**Fig. 1:**
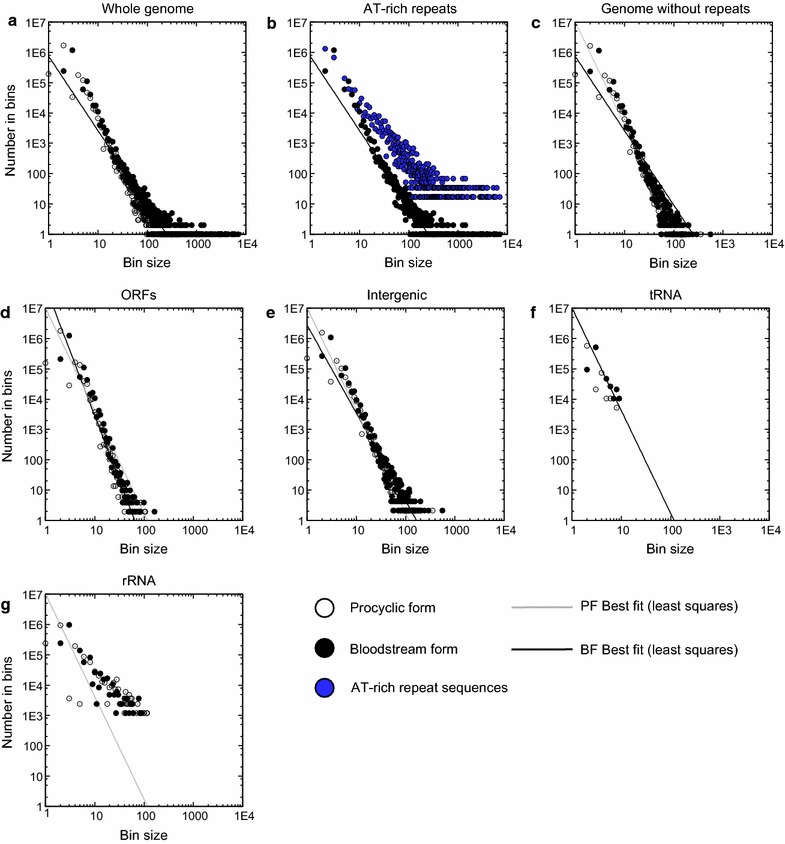
Binning analysis of nucleosome dyad co-alignment in the *T. brucei* genome. Binning analysis allows the visualization of nucleosome density and positioning in different genomic regions. The bin size (*x axes*) indicates the number of co-aligned nucleosome dyads reflecting nucleosome positioning strength, and the number of members in each bin (*y axes*) represents the number of times this degree of positioning was observed in the tested genomic region. **a** These analyses were performed on the entire *T. brucei* 427 genome using chromatin from either BF or PF. **b** Nucleosome distribution in the whole genome compared with AT-rich repeat sequences in BF chromatin. **c** Comparison of chromatin from BF and PF genomes excluding AT-rich repeat sequences. **d** Nucleosome distribution at the coding regions in BF and PF chromatin. **e** Comparison of nucleosome positioning at intergenic regions (SSRs as well as noncoding regions between individual genes in a PTU) in BF and PF *T. brucei*. **f** Nucleosomes at *tRNA* genes in BF and PF. **g** Nucleosomal distributions at the *5S* subunit *rRNA* genes in BF and PF. The *lines* represent the best fit (*least squares*) line to the BF (*black line*) or PF (*gray line*) datasets, respectively

In eukaryotic chromatin, approximately 147 nucleotides (nt) of DNA are wrapped around each histone octamer. Digestion of this chromatin with micrococcal nuclease (MNase) results in release of these 147-nt fragments, which if sequenced using high-throughput methods, allows nucleosome positioning over the entire genome. We therefore mapped nucleosomes at the whole genome level in the *T. brucei* 427 BF or PF life cycle stages using MNase-seq. This involved mapping the paired-end reads of isolated nucleosomal fragments of ~147 bp to the *T. brucei* 427 reference genome. Nucleosome dyad positions were assumed to correspond to the center of the mapped fragments. To gain insight into the distribution of nucleosomes in the genome of *T. brucei*, we performed a binning analysis, which makes it easier to visualize nucleosome density and positioning in different genomic regions. Here we summed the number of times that specific dyad frequencies were observed in the genome, using bin values from 1 to the maximum observed dyad frequency (Fig. 1a). The bin size represents the number of co-aligned nucleosome dyads, reflecting nucleosome positioning strength, and the number of members in each bin represents the number of times this degree of positioning was observed in the tested genomic region. Nucleosomes that aligned with AT-rich repeats, which are also present in intermediate- and mini-chromosomes not included in the current *T. brucei* reference genome, were also masked to avoid copy-number effects (Fig. 1b, c).

There was not a significant difference in nucleosome density between the BF and PF life cycle stages, and coding sequences contained 0.22 and 0.21 dyads/bp in both BF and PF *T. brucei*, respectively (Fig. 1d). The intergenic regions (SSRs as well as noncoding regions between individual genes in a PTU) had a nucleosomal dyad density of 0.22 dyads/bp in both the BF and PF life cycle stages (Fig. 1e). A slight extension of the bin population to higher value bins (or more well-positioned nucleosomes at a given genomic region) was visible in the intergenic, compared to the coding regions (compare Fig. 1d, e). This increase is statistically significant (Whitney–Mann *U* test; *p* < 0.001), showing that highly positioned nucleosomes were present more often in intergenic regions. No statistically significant difference could be detected between the distribution of nucleosomes in BF and PF forms in either the coding sequences or intergenic regions (Whitney–Mann *U* test; *p* > 0.05). This result suggests that the bulk nucleosome density and proportion of well-positioned nucleosomes were comparable in BF and PF *T. brucei*.

In the case of the predominantly pol III-transcribed *tRNA* genes, an average dyad density of 0.1 dyads/bp was found, with a normalized bins distribution as shown in Fig. 1f. The absence of high-value dyad bins indicates that nucleosomes are generally weakly positioned on the *tRNA* genes, which may be related to the transcriptional activity of *tRNA* genes and the size of the pol III transcription complex compared to the size of the *tRNA* gene itself. In yeast, *tRNA* genes are occupied by the TFIIIB–TFIIIC complex, displaying a distinctive occupancy pattern termed a “bootprint,” and are generally nucleosome free [48]. A TFIIIC ortholog has not been identified in *T. brucei*, and it is not clear whether any transcription-related protein complex binds to the putative A-box [49]. *rRNA* genes had a dyad density of 0.34 dyads/bp and accommodated more well-positioned nucleosomes (Fig. 1g) compared to the genome average (see Fig. 1c; indicated by the gray diagonal in Fig. 1g). This may indicate the presence of a subpopulation of inactive *rRNA* genes, which is a common feature of the rDNA transcription units in other eukaryotes [50], assuming the number of *rRNA* genes annotated in the reference genome is accurate. The bulk nucleosome repeat length, or average nucleosome dyad to dyad distance, was determined at 194 bp in both the BF and PF life stages (data not shown).

### *5S rRNA* genes

**Fig. 2:**
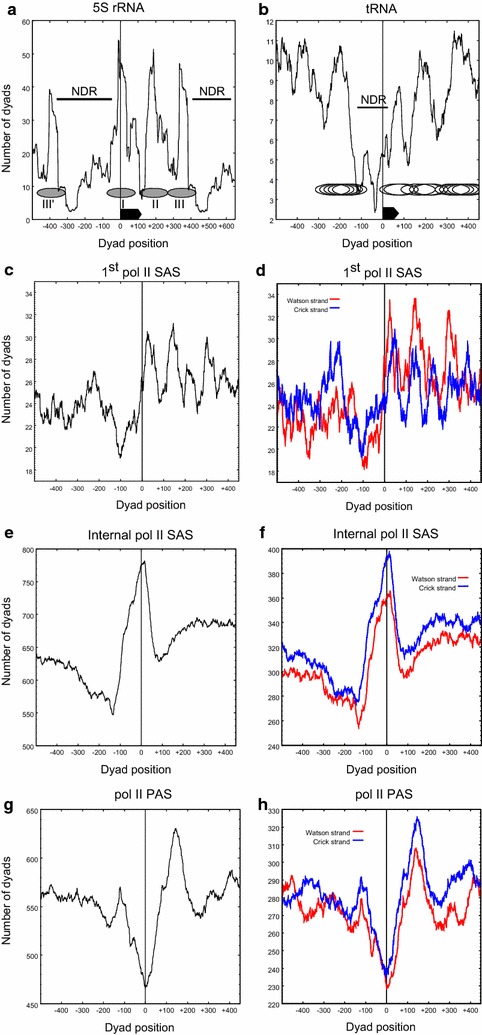
Alignment of nucleosomal dyads at pol I-, II- and III-transcribed loci in BF trypanosomes. The number of nucleosome dyads assigned to each nucleotide was summed for all aligned features. Genes were aligned relative to the TSS, SAS or PAS, indicated by the *vertical black line* at dyad position 0 in each panel. **a** The average, cumulative nucleosomal dyad distribution is shown for *5S rRNA* genes and **b** for *tRNA* genes. The extent of the 119-bp *5S rRNA* (**a**) and 74-bp *tRNA* (**b**) genes are shown by the *black*, *rectangular arrows*. The ellipses **a**, **b** indicate the span of 160-bp nucleosomes, with the ellipse centers (nucleosomal dyads) aligned to the center of the corresponding, major peaks. The nucleosomes in **a** are labeled I–III, and the III′ and III nucleosomes indicate identical nucleosomes in the repeating *5S* array. **c** Alignment relative to the SAS of the first coding sequence of all PTUs. **d** Separate alignment of the Watson and Crick strand data relative to the SAS of the first coding sequence of all PTUs. **e** Alignment relative to the SAS of all coding sequences within all PTUs. **f** Separate alignment of the Watson and Crick strand data relative to the SAS of all coding sequence within all PTUs. **g** Dyad axes aligned relative to the PAS of all genes in all PTUs. **h** Dyad axes aligned to the Watson and Crick strand data relative to the PAS of all genes within all PTUs

The pol III-transcribed *5S rRNA* genes are arranged as a single cluster from position 454,171–460,512 on chromosome 8 with a spacing of approximately 620 bp between each 118-bp *5S* gene. When the nucleosomal distribution of each *5S* gene was aligned relative to the transcript start position (Fig. 2a), defined as the first nucleotide in the *5S rRNA* gene sequence, a very clear nucleosomal organization emerged. Three well-positioned nucleosomes were seen on the *5S* transcription unit, with the first nucleosome centered on the transcript start. The downstream two nucleosomes were positioned directly downstream of the transcript end (represented by the ellipses in Fig. 2a). Nucleosome I overlapped with the *5S rRNA* gene up to approximately position 80 of the 5S transcript, which would include the putative A box (involved in pol III transcription initiation) located at position 51–62 of the gene (Additional file 1: Fig. S1).

A nucleosome-depleted region (NDR) was observed upstream of the *5S rRNA* gene, preceded by a well-positioned nucleosome at approximately position −400, which was equivalent to the second downstream nucleosome in the repeating 5S unit (labeled III′ and III, respectively, in Fig. 2a). The eight *5S rRNA* genes in the *T. brucei* 427 reference genome encode transcripts that are 100% identical, and the 620-bp intergenic regions are 98% identical at nucleotide level. The alignment of sequence reads to the reference genome was therefore averaged between the individual genes by the mapping procedure. The distribution of the 3 identified nucleosomes was at sterically allowed distances (nucleosome I–II, 185 bp, and nucleosome II–III, 160 bp). There was no detectable difference in the nucleosomal organization of the *5S* genes in the BF and PF life cycle stages (Fig. 2a; Additional file 1: Fig. S2A).

*Trypanosoma brucei* 427 contains two larger *rRNA* gene clusters with 24% identity at the nucleotide level located on chromosomes 2 and 3, and six smaller clusters of *rRNA* genes on chromosomes 6, 7, 8, and 9. The small number of annotated *rRNA* gene clusters precludes an informative alignment of the nucleosomal dyads at these loci.

### *tRNA* genes

We next aligned the 64 *tRNA* encoding genes at the transcript start positions. A clear NDR was again discernible directly upstream of the *tRNA* genes (Fig. 2b). This region was bracketed by groups of nucleosomes that appear to be located in multiple, overlapping frames. Three groups of nucleosomes were typically visible downstream of the *tRNA* gene and may represent three abutting nucleosomes in multiple phases. Upstream of the *tRNA* gene another group of overlapping nucleosome positions was typically visible. The nucleosomal arrangement at the *tRNA* genes appears functionally important, since the NDR, and the single upstream and three downstream nucleosomes were independently visible on the Watson and Crick strands (Additional file 1: Fig. S2B; Kendall’s tau correlation; *τ* = 0. 56, *p* < 0.001), suggesting that they were arranged relative to the direction of transcription of the *tRNA* gene. However, the nucleosomal dyad distributions shown in Fig. 2b represent the contribution from both active and inactive genes, and the first downstream nucleosome may be unstable or disrupted in active *tRNA* genes, thereby not contributing substantially to the dyad distribution.

### Nucleosomal organization of pol II PTU transcription initiation regions

In other eukaryotes, a general picture has emerged for nucleosomal organization associated with the upstream regions of pol II-transcribed genes, where well-positioned nucleosomes flank an NDR that largely overlaps with the TSS [14]. This NDR is thought to allow assembly of the pol II pre-initiation complex in the region of the TSS [14]. However, in *T. brucei* pol II-mediated transcription is unusual in the sense that conventional pol II promoters are absent or undefined, and transcription commences at divergent SSRs and at a few other unique non-SSR locations [51]. Sequence analysis has failed to identify conventional TATA boxes or initiator sequences in the pol II transcription start regions [52], and it is thought that *T. brucei* lacks the many transcriptional activators, basal factors and *cis*-elements associated with pol II gene expression in eukaryotes from the Unikonta supergroup [53]. Although the pol II transcription start region is enriched for the H2A.V and H2B.V histone variants, and epigenetic marks associated with transcriptional activation in model eukaryotes [42], it is not understood how these histone variants and modifications are targeted to these precise chromatin regions. We were, therefore, interested in the nucleosomal organization of these pol II transcription start regions, and the idea that the local nucleosomal landscape may define functional pol II TSSs in *T. brucei*.

As the pol II TSSs remain unmapped in *T. brucei*, we aligned the nucleosomal dyads of PTUs relative to the most upstream splice acceptor site (SAS) which was identified in a *T. brucei* transcriptomic study [51]. The SAS is located upstream of each coding sequence in a PTU and is where the 39-nt sequence spliced leader RNA is *trans*-spliced onto the nascent RNA. The SAS is functionally related to the 3′ end of introns at intron–exon junctions in metazoans [54]. In the case of the SAS of the first gene of the PTU, this would be the genomic feature that is closest to the pol II transcription initiation region. When aligned relative to the first SAS (Fig. 2c), a region that was weakly depleted of nucleosome dyads was visible. This region was depleted of nucleosomal dyads in an approximately 100-bp region upstream of the SAS of the first gene of the PTU on both the Watson and the Crick strand (Fig. 2d), suggesting that this nucleosomal organization was sensitive to the direction of transcription (Kendall’s tau correlation *p* < 0.001).

Although evidence of positioned nucleosomes downstream of the NDR was visible, these nucleosomes did not form a single phased array relative to a defined genomic feature, such as a +1 nucleosome, as is seen in other eukaryotes [14, 55, 56]. The observed peaks were less than the allowed nucleosome-to-nucleosome distance, indicating the absence of a regularly spaced nucleosome array, in both BF and PF cell lines (Additional file 1: Figs. S3 and S4). Although this nucleosomal arrangement is reminiscent of nucleosomes that were mapped downstream of the pol II TSSs in *S. cerevisiae* [55] and *Drosophila* [56], we note that the pol II PTUs in *T. brucei* are constitutively expressed, and a well-defined nucleosomal array in this region would be unexpected. A similar pattern of nucleosomal distribution surrounding pol II TSS has been observed in a related kinetoplastid, *Leishmania major* [57]. A high nucleosome occupancy (defined as the average nucleosome dyad density per base pair in a specified region), but little positioning (defined as the number of co-aligned dyads at a specific base pair), was observed across the constitutively transcribed pol II PTUs [57]. The nucleosomes downstream of the first SAS appeared less organized in the PF life cycle stage (Additional file 1: Fig. S4A–D). In our experiments, we did not partially crosslink the nucleosomes chemically. However, the less-ordered nucleosomal arrangement in these regions is unlikely to be due to nucleosome sliding, since we do observe stable and strongly positioned nucleosomes at the *5S rRNA* genes.

These results showed that a region displaying a weak nucleosomal depletion was present upstream of the first SAS of pol II PTUs in *T. brucei*. This NDR was not as well defined as that seen at pol II TSSs in other eukaryotes such as in *S. cerevisiae* [14], and its functional association with the TSS, which remains unmapped in *T. brucei*, is also uncertain. If this NDR is indeed linked to transcription initiation, it suggests that this organizational feature, probably inherited from an Archaeal ancestor, was not maintained to the same extent in *T. brucei* compared with other eukaryotes. A clear enrichment of the H2A.Z histone variant was seen in nucleosome +1 bordering the NDR at the pol II TSSs in eukaryotic model organisms from the Unikonta supergroup [14]. The structurally less stable H2A.Z-containing nucleosome was proposed to facilitate elongation by pol II [58]. We therefore mapped H2A.V ChIP-seq data from *T. brucei* (kindly made available by N. Siegel) to our MNase-seq data. Although a clear enrichment in H2A.V-containing nucleosomes was seen in the upstream regions of PTUs and overlapping with the first gene, as was previously reported [42], this broad enrichment of nucleosomes did not map to any single, well-positioned nucleosome (data not shown).

### The NDR is not a feature of all SAS regions

We next assessed whether the nucleosomal organization observed for the region surrounding the SAS of the first gene in pol II PTUs was specific to this region, or simply reflected the nucleosomal organization of all upstream SAS regions for all genes within a PTU. We therefore repeated the dyad alignment analysis for all genes with assigned SAS sites, excluding that of the first gene in a PTU (3178 genes). The result is shown in Fig. 2e. In stark contrast to the arrangement seen at the SAS upstream of the first gene in a PTU, a striking enrichment of nucleosomal dyad axes that were aligned with the mapped SAS was seen. This was observed in the biological duplicates in both cell lines of the BF and PF life cycle stages (Additional file 1: Fig. S5) and was also independently seen (Kendall’s tau correlation *p* < 0.001) on the assigned SAS of both the Watson (1545 genes) and Crick (1633 genes) strands (Fig. 2f). This suggests that there is a well-positioned nucleosome preferentially aligned with each internal SAS in *T. brucei*, contrary to the NDR observed at internal SASs in *L. major* [57]. When studying the nucleosomal arrangement at the SAS at the level of individual genes, the consistent, tight positioning of a nucleosome in the direct vicinity of the SAS is often observed, with a more random distribution of nucleosomes in the regions adjacent to the SAS (Additional file 1: Fig. S6). In contrast, bordering nucleosomes appeared disorganized.

### Nucleosomal organization at pol II termination regions

In addition to the SAS, the individual genes in a PTU typically contain a 3′ UTR with an average length of 676 bp, terminating at the polyadenylation site (PAS) [51]. The genomic region downstream of the PAS of the last gene of the PTU does not appear to contain defined transcription termination sequences [59]. The pol II transcription termination regions are enriched for the histone variants H3.V and H4.V [42], and both H3.V and base J deposition in this region appear to act synergistically to mediate efficient pol II termination [46, 60]. We were, therefore, interested in possible specialized nucleosomal arrangements in the regions of pol II transcription termination.

We first aligned the nucleosomal dyad axes relative to the PAS elements of all genes within PTUs [51] to get an overview of the general PAS structure. In contrast to the average nucleosomal organization around the internal SAS element, the PAS was clearly depleted of nucleosomes, with a single well-positioned nucleosome present immediately downstream of the PAS (Fig. 2g). Again, similar nucleosomal arrangements were independently observed on the Watson (1364 genes) and Crick (1448 genes) strands (Kendall’s tau correlation; *τ* = 0.46, *p* < 0.001, Fig. 2h), suggesting that the nucleosomal arrangement was important to a directional process on the DNA molecule (presumably transcription). This nucleosomal arrangement is very similar to that seen in human and yeast genomes, where nucleosomes are also depleted on polyadenylation sites [61, 62]. A similar organization was also seen in *L. major*, although the observed NDR was present immediately upstream of the PAS [57]. However, the relatively small number of terminal PAS elements (*n* = 35) precluded any statistically meaningful analysis of the average nucleosomal organization at this genomic position.

### Genomic distribution of nucleosome refractory sequences

It has previously been shown that oligo-d(A·T) runs are generally excluded from central locations of isolated chicken nucleosome cores [63], and it was suggested that this depletion is due to the inherently rigid structure of A·T tracts due to a series of bifurcated hydrogen bonds [64]. In *T. brucei*, runs of oligo-d(A·T) of 7 bp and longer are present in nucleosomes at approximately 70% of that expected for a random distribution (Additional file 1: Fig. S7A). Interestingly, runs of oligo-d(G·C) of up to 4 nucleotides were present in nucleosomes more often or the same as that expected from a random distribution, with a striking absence of runs longer than 7 bp (Additional file 1: Fig. S7B). Oligo-d(A-T) and oligo-d(T-A) are markedly depleted in *T. brucei* nucleosomes (Additional file 1: Fig. S8), even though these sequence runs occur at very high frequencies in the genome (Additional file 1: Table S1). This depletion might possibly be due to the destruction of nucleosomal fragments containing these sequences during nucleosome core preparation and subsequent rarefaction in the sequencing sample. It therefore appears that runs of oligo-d(A·T) and oligo-d(G·C) contribute to the relative absence of nucleosomes in specific regions of the *T. brucei* genome.

**Fig. 3:**
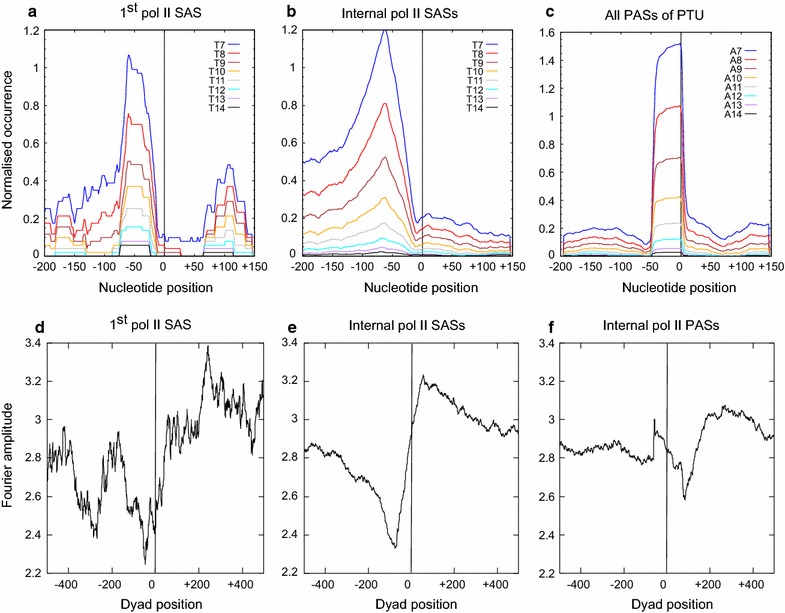
Distribution of nucleosome positioning signals at SASs and PASs. **a** The sequences of 400-bp regions encompassing the SAS (where it has been annotated [51]) were retrieved for the first gene in all PTUs, **b** for all internal SASs, and **c** for all PASs in all PTUs. The number of oligo-dT or oligo-dA runs (7–14 bp) was determined for the sequences aligned at the annotated SASs and PASs and is shown as a value normalized to the number of sequences. The Fourier amplitude of the distribution of A–A dinucleotides at a 10-bp periodicity was determined in a sliding 128-bp window, and normalized to the number of sequences in the window. **d** The cumulative Fourier amplitude is shown in a range from −500 to +500 bp relative to the first SAS upstream of all PTUs, **e** relative to internal SASs in all PTUs, and **f** relative to the internal PAS in all PTUs. The location of the SAS or PAS is indicated by the *vertical black line*

The polycistronic nature of the PTUs in *T. brucei* requires a SAS upstream of each open reading frame (ORF) to allow the *trans*-splicing of the 39-nt spliced leader (SL) RNA. The 3′ splice acceptor site contains the AG dinucleotide and a polypyrimidine tract (PPT) which is typically 10–40-nt upstream of the SAS, and is recognized by a U2AF35 and U2AF65 heterodimer of the spliceosome in the pre-mRNA [65]. As we have found that oligo-d(A·T) and oligo-d(G·C) runs are underrepresented in nucleosomes, we wondered whether the PPTs upstream of the SAS of each gene, and in particular the first gene of a PTU, was involved in the structural organization of nucleosomes in these regions. The preference for oligo-dT, as opposed to oligo-dA on the coding strand, is a requirement of the splicing mechanism [66]. Looking at the distribution of T runs of 7 bp and longer, a clear concentration of such runs is visible upstream of the first SAS (Fig. 3a) as well as all internal SASs (Fig. 3b). A second region enriched for oligo-dT appears downstream of the first SAS in a region that mostly falls within the 5′ UTR of the RNA transcripts. This second region of oligo-dT enrichment is absent in the average distribution of T runs at internal SASs (Fig. 3a, b).

The nucleosomal organization in the region of the first SAS of a PTU compared to the average organization of all PTUs differed significantly (see Fig. 2c, e). A region weakly depleted of nucleosomes was observed upstream of the first SAS (Fig. 2c, d), and a well-positioned nucleosome was observed at all internal SAS sites (Fig. 2e, f). The region of nucleosome depletion at position −50 to −150 does not precisely align with the region enriched for T·A runs at position −70 to −20. However, the overlap in these regions, and the observation that oligo A runs in excess of 7 bp are generally depleted of nucleosomes, makes it highly likely that the presence of the oligo-d(A·T) runs upstream of the SAS contributes to the appearance of an NDR. However, the positional mismatch between the oligo-d(A·T) run and the NDR suggests the involvement of additional factors in establishing the NDR. The second region of oligo-dA enrichment at position +100 relative to the SAS of the first gene may serve to position the first nucleosome of the first gene of a PTU (Fig. 2c, d). The absence of this second oligo-dA-enriched area at the remaining genes in a PTU may contribute to the absence of positioned nucleosomes downstream of the nucleosome positioned on the SAS (Fig. 2e, f).

A striking enrichment of oligo-dA runs is present directly upstream of the PASs (Fig. 3c), partially overlapping with the NDR observed in this region (Fig. 2g). In higher eukaryotes, a highly conserved AATAAA hexanucleotide that signals polyadenylation is located upstream of the PAS and may be refractory to nucleosomes [62]. However, this *cis*-acting element seems to be absent in *T. brucei* [67] and the role of the observed oligo-dA run abutting the PASs is unclear, but might contribute to the observed NFR at PASs.

### Nucleosome positioning signals at the initial SAS, internal SASs and internal PASs

Travers and colleagues showed that the rotational position of isolated nucleosome cores is defined by a distribution of dinucleotides at a periodicity equal to that of the DNA helix [63]. This was interpreted in terms of the structural constraints imposed on the rotational freedom of specific dinucleotide steps, and the ability to accommodate a narrowed or expanded minor groove. It was subsequently shown in genome-wide studies that positioned nucleosomes were often associated with di- and trinucleotide distributions equal to the DNA periodicity [55]. We therefore investigated whether the positioned nucleosomes upstream of the first gene in a *T. brucei* PTU, as well as those present at internal SASs and at the PASs, was positioned by underlying sequence periodicity.

We analyzed the distribution of the A–A dinucleotide by plotting the Fourier amplitude in a 128-nt window at consecutive settings across sites of interest in the genome, showing the strength of a periodic distribution of a given nucleotide. In the case of the first gene of a PTU, the highest Fourier amplitude is seen at approximately position +200 bp (Fig. 3d), indicating the likely presence of a strong nucleosome positioning sequence in this region. This aligns with the nucleosome immediately downstream of the NDR (see Fig. 2c) of the first SAS in a PTU. Interestingly, this nucleosome fits into the region forming a saddle between the two peaks of enrichment for oligo-dA (Fig. 3a), and the NDR (Fig. 2c), and partially overlaps with the peak at −50 in the distribution of oligo-dA (Fig. 3a). Therefore, the nucleosome distribution in the region of the SAS upstream of the first gene of a PTU can be explained in terms of the enrichment and depletion of specific sequence elements in this region and the known preference of nucleosome cores for such elements.

When looking at the internal SASs, a region generally enriched for 10-bp A–A periodicities is seen downstream of the SAS (Fig. 3e). Upstream of the SAS, centered at approximately position −80 bp, a region depleted of 10-bp A–A periodicities is evident. This aligns almost perfectly with the region enriched for oligo-dA tracts (Fig. 3b). The average nucleosomal structure in the region showed a nucleosome positioned at approximately position 0 (Fig. 2e, f). Thus, this nucleosome position is also consistent with the distribution of 10-bp A–A periodicities and oligo-dA tracts in these regions.

Looking at the Fourier amplitude of all annotated PASs (Fig. 3f), a region slightly enriched for a 10-bp AA periodicity is seen downstream of position +150. Comparing this distribution to that of oligo-dA runs (Fig. 3c), a strong peak of oligo-dA runs is typically seen directly upstream of the PAS. Thus, there appears to be a sequence arrangement that discourages nucleosome formation from position 0 of the PAS and is more facilitative of nucleosome deposition downstream of the PAS. This is exactly what was seen in the average nucleosomal organization surrounding the PAS elements (Fig. 2g, h), where position 0 was depleted of nucleosomes, and a strongly positioned nucleosome was visible centered at approximately position +150. Again, the nucleosomal organization can be explained in terms of the sequence elements present at the PAS.

It was previously shown that nucleosome positions in vivo are directed by sequences as well as DNA binding proteins that may initiate “statistical positioning,” as well as by chromatin remodelers [68]. Our results do not exclude the contribution from agents other than sequence, and the NDR upstream of the first SAS of a PTU cannot be fully attributed only to the polypyrimidine tract, which overlaps only partially with the NDR, thus implying the involvement of other factors.

### Nucleosome organization in BF and PF *T. brucei* life cycle stages is highly comparable

There is little evidence for pol II transcriptional control in *T. brucei*, and the life-cycle-specific control of most genes occurs posttranscriptionally [40, 69, 70]. However, some pol I-transcribed loci are differentially transcribed in the different *T. brucei* life cycle stages. For example, only one of about 15 VSG expression sites is active in BF *T. brucei*, whereas all ESs are repressed in the procyclic form. In contrast, the procyclin genes are repressed in the BF and active in the PF stage. We were therefore interested in establishing whether there were any clear differences in the nucleosomal organization of the *T. brucei* genome in the BF or PF life cycle stages that could explain this differential expression.

**Fig. 4:**
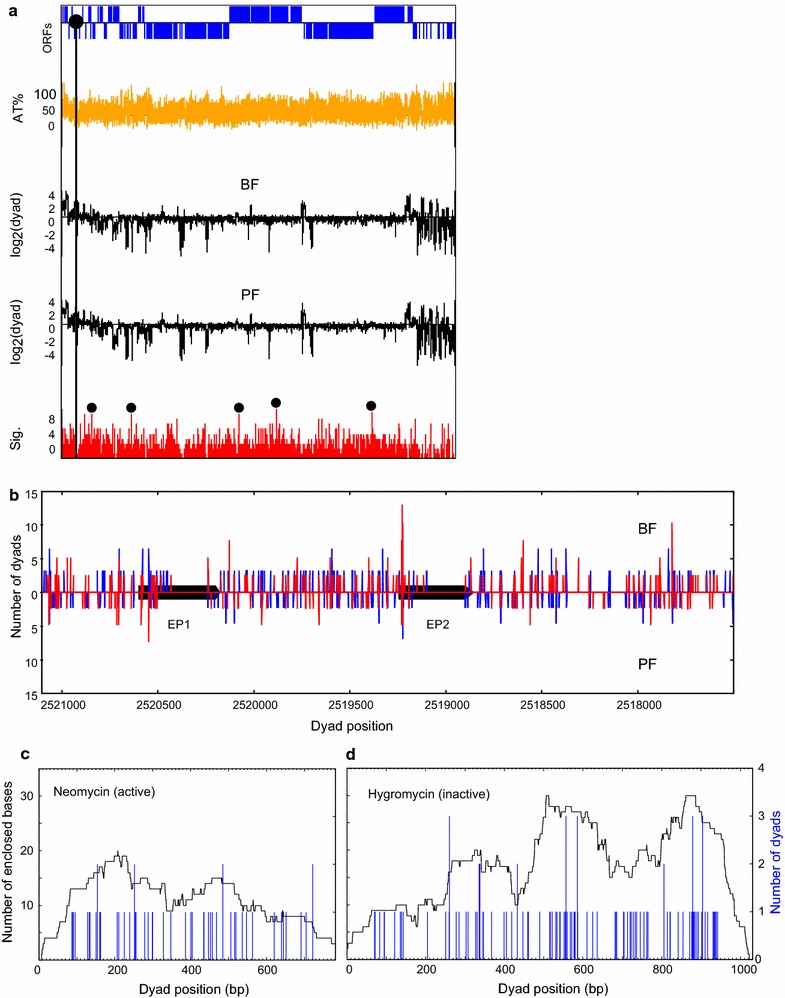
Genome-wide nucleosomal organization in BF and PF *T. brucei*. **a** Chromosome 6 is selected as example, and statistically significant differences between BF and PF stages are shown. The *top line* indicates the position of assigned genes on the Watson strand (*above horizontal axis*) and the Crick strand (*below horizontal axis*). The location of the centromere is indicated by the *filled circle*. The *yellow line* shows the A/T % at each setting of a 100-bp scanning window. The *black traces below* show the traces of the log_2_ ratio of the average number of assigned dyad axes in a 1000-bp scanning window to the genome average in either BF or PF. The correlation between the relative nucleosome density in BF and PF chromatin was calculated by the Whitney–Mann *U* test at 50-bp intervals in a 500-bp sliding window. The number of statistically significant (*p* < 0.01) samples in four biological replicates was added and is shown as a “significance” value in the *red traces*. The *small black circles* identify peaks of high significance where the nucleosomal organization of the corresponding regions was individually assessed. **b** The distribution of dyads at the *EP1*–*EP2* procyclin locus (*black bars*) in a 3500-bp region. In each case, the *red* and *blue impulse* plots represent two biological replicates of BF (*top panel*) and PF (*bottom panel*) chromatin. **c**, **d** Alignment of nucleosomal dyads in chromatin from BF HNI_VO2 cell line. Coding sequences of the neomycin and hygromycin single copy resistance marker genes, present immediately downstream of the pol I ES promoters, were analyzed. Lower levels of well-positioned nucleosomes were present on the active neomycin gene (**c**), with regions of dyad enrichment observed for the transcriptionally inactive hygromycin gene (**d**). *Y*-axes scales are mirrored, with the *left-hand y-axes* indicating number of enclosed bases (*black trace*), and the *right-hand axes* indicating number of dyads (*blue*)

The average density of nucleosomal dyads in the *T. brucei* genome was determined, and the ratio of the number of dyads in a 1000-nt window compared with the genome average was plotted on a log_2_ scale. Chromosome 6 was chosen as a representative (Fig. 4a; all chromosomes are shown in Additional file 1: Fig. S9). A superficial inspection did not reveal any striking differences in the nucleosome occupancy traces on any chromosomes when comparing BF versus PF *T. brucei* (Fig. 4a, Additional file 1: Fig. S9). However, to quantitate possible subtle differences, we performed a Whitney–Mann *U* correlation analysis. Statistically significant (*p* < 0.01) differences between the two life cycle stages are shown as the lower red trace for each chromosome (Fig. 4a). The five most significant peaks for each chromosome were chosen, and the corresponding region in the genome was further investigated. The regions with different nucleosome occupancy did not map to any predominant functional feature. Identified differences were located within and between PTUs, on coding and intergenic regions, and were not correlated with classes of genes that were relevant to a specific life cycle stage. This suggests that the identified differences in nucleosomal occupancy were probably not meaningful.

We next investigated the chromatin structure of transcription units which are expressed in a life-cycle-specific fashion. The *EP1* and *EP2* procyclin genes are not expressed in BF *T. brucei*. A larger number of nucleosomal dyads were present at these loci in BF compared with the PF *T. brucei* (Fig. 4b) and extended further downstream into the procyclin coding sequence in the BF cells. This indicated that the *EP1* and *EP2* procyclin genes have structurally more compact chromatin in BF cells, possibly impeding transcription of these procyclin loci in BF, compared with the PF cells. ESs are only transcribed at a high rate in BF *T. brucei*. Unfortunately, the high degree of sequence identity of the 15 ESs precluded unique read mapping and the analysis of nucleosome organization at active and inactive ESs. However, the BF HNI_VO2 cell line contains unique sequences in the form of neomycin and hygromycin resistance genes in the active and inactive ESs, respectively. Looking at the nucleosomal distributions on these single copy, unique sequence markers, fewer well-positioned nucleosomes were present on the active drug resistance gene compared with the inactive one (Fig. 4c, d). In fact, 0.07 (53 dyads per 782-bp gene) dyads were assigned per base pair in the case of the transcriptionally active neomycin gene, as opposed to 0.11 (109 dyads per 1031-bp gene) dyads per base pair in the case of the inactive hygromycin gene. Although this is in agreement with previous observations [71, 72], it is not clear to what extent sequence differences contributed to this difference in nucleosome occupancy. In summary, subtle differences in the nucleosomal organization of life-cycle-specific genes were observed, which could be an effect of active transcription. However, we saw no difference in the organization of chromatin over extensive genomic regions between the two *T. brucei* life cycle stages (Fig. 4a; Additional file 1: Fig. S9).

### Nucleosomal organization at the silent *VSG* arrays

**Fig. 5:**
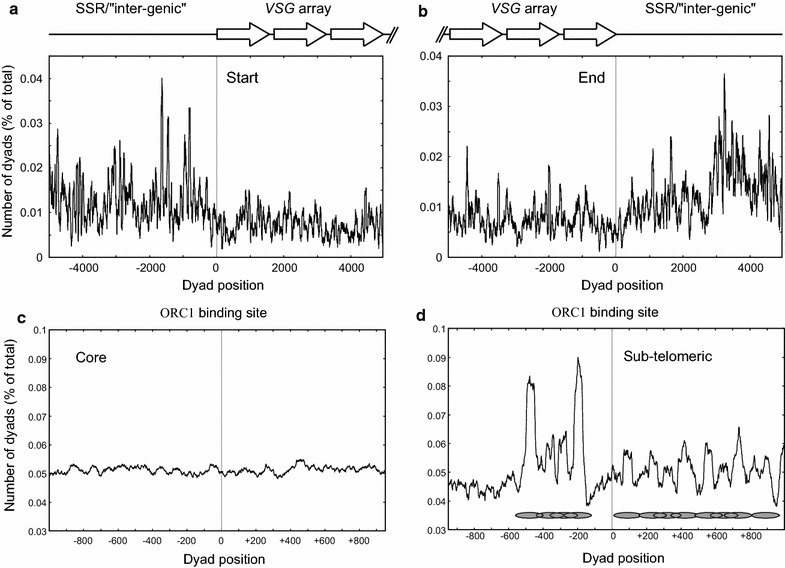
Nucleosomal organization around the silent *VSG* gene arrays and DNA origins of replication. Nucleosome dyads were analyzed in a 10-kb region at the beginning or end of arrays of silent *VSG* genes located at the right-hand side of chromosome 9. **a** These dyads were analyzed within a 10-kb region at the beginning of VSG arrays (18,449 dyads), or **b** at the ends of these arrays of silent *VSG* genes (21,824 dyads). These beginning and end points are indicated by the *vertical gray lines* in **a**, **b**. The alignments of the regions between adjacent blocks of co-aligned *VSG* genes (SSR/“intergenic”) and the *VSG* genes and pseudogenes (*VSG* array) are schematically shown above *each panel*. **c** The distribution of nucleosomal dyads located in the chromosomal core regions of the *T. brucei* genome (434,752 dyads). The *gray line* represents the position of the center of the ORC1 binding site. The number of nucleosomal dyads present at each nucleotide position is expressed as a percentage of the total number of dyads in the analysis. **d** The distribution of nucleosomal dyads in the subtelomeric region (72,616 dyads) was aligned relative to the center of mapped ORC1 binding sites [74]. The alignment of nucleosomes with major nucleosomal dyad peaks is shown as *gray ovals*, with the telomere end located at the *right of the panel*

*Trypanosoma brucei* encodes a large number of transcriptionally silent *VSG* genes and pseudogenes present in large tandem arrays on chromosomes 5, 9 and 11. In addition, silent *VSGs* are present immediately at the telomeres of all classes of chromosomes [22]. We investigated whether the silent *VSG* arrays have a defined nucleosomal organization, with specific bordering structures. This could represent possible silencing elements, as seen at the silent mating-type loci in *S. cerevisiae* [73]. We therefore investigated the chromatin structure around these silent *VSG* arrays. The nucleosomal dyads were aligned in 10-kb regions relative to the first nucleotide on the most upstream gene of a block of tandem *VSG* genes (“start”), or the last nucleotide of the most downstream gene in a block of tandem *VSG*s (“end”), and are shown in Fig. 5. It is immediately clear that the silent *VSG* arrays on chromosome 9 are enclosed by regions of well-positioned nucleosomes, both upstream of the “start” (Fig. 5a) and downstream of the “end” (Fig. 5b). The silent *VSG* arrays themselves are clearly packaged into nucleosomes in both BF and PF *T. brucei*, although the positions are less defined compared with the flanking regions. A representative organization of one of these *VSG* arrays in BF cells is shown in Fig. 5. Similar clusters of well-positioned nucleosomes were seen bordering silent *VSG* arrays on the left-hand side of chromosome 9 as well as on chromosomes 5 and 11.

The observed well-positioned nucleosome structures flanking the silent *VSG* arrays could result in repressing fortuitous transcription initiation of these silent *VSGs*, thereby maintaining mono-allelic expression of the active *VSG*. The more poorly positioned nucleosomes covering the *VSG* arrays may represent an open chromatin structure more amenable to DNA recombination events, or may be due to a repressive chromatin structure resulting in decreased MNase cleavage.

### TbORC1 and DNA replication origins

It had previously been shown that ORC1, a component of the origin recognition complex, binds to numerous regions in the *T. brucei* genome, many of which act as origins of DNA replication [74]. It has also previously been shown in *S. cerevisiae* that ORC1, apart from its role in DNA replication, is also a component of silencing complexes assembled at silencing elements such as the E-element of the Mat alpha silent mating-type locus. Consequently, we were interested in establishing whether the *T. brucei* ORC1 was similarly involved in a specialized nucleosomal organization which could be implicated in gene silencing.

We mapped ORC1 sites identified in *T. brucei* 927 [74] to the equivalent sequences in the genome of *T. brucei* 427. The ORC1 binding sequences originally identified by Tiengwe and colleagues [74] ranged from 65 bp to 3 kb. This mapping was therefore at a low resolution, below that of single nucleosomes. Nevertheless, we utilized this dataset to investigate the nucleosomal organization in the vicinity of assigned ORC1 binding sites.

We first investigated the DNA region surrounding ORC1 sites in the core region of chromosomes (994 sites), which contain the constitutively expressed housekeeping genes. Here, very little nucleosomal organization relative to the center of the assigned DNA replication origin was discernible (Fig. 5c). We next investigated the subtelomeric regions (119 sites), which are defined as regions adjacent to telomeric ends. These contain the silent *VSG* gene and pseudogene arrays, expression site-associated genes (*ESAG*s), and the highly repetitive retrotransposon hotspot proteins (*RHS*) gene family. Here, a very clear nucleosomal organization was evident (Fig. 5d). The differences in organization of nucleosomes within the coding and flanking regions of the silent *VSG* arrays are consistent with the presence of specialized bordering silencing complexes, as also suggested by McCulloch and colleagues [72]. Intriguingly, of the 15 high confidence (false discovery rate (FDR) <0.05; [74]) ORC1 binding sites identified in the subtelomeric region of chromosome 9, 14 were present in the silent *VSG* array. This result strongly suggests that ORC1 binding sites, and presumably ORC1 itself are involved in the demarcation of specialized chromatin domains associated with silencing of the *VSG* gene and pseudogene arrays, as is the case in *S. cerevisiae* silent mating-type loci [73]. This suggests that the role of ORC1 in arranging surrounding chromatin structure and repressing regional transcription is evolutionarily ancient, and was likely present in LECA.

## Discussion

*Trypanosoma brucei* regulates the expression of most of its protein-coding genes at the posttranscriptional level. However, the *T. brucei* genome encodes homologs for putative chromatin writers, readers and erasers, as well as chromatin remodeling enzymes and histone variants [3–6]. It has been shown that specific histone variants demarcate the borders of PTUs [42]. In addition, various epigenetic players were shown to be important in the regulation of the mono-allelic expression of the active *VSG* ES, and the concomitant repression of the approximately 14 silent ESs [28, 33, 36, 37, 42, 44, 71, 74–76].

In this study, we present a whole genome analysis of nucleosomal positioning in *T. brucei*. We provide clear evidence for locally organized nucleosomal structures in both BF and PF *T. brucei*. A general feature of the transcription initiation region of pol II-transcribed genes in model organisms from the Unikonta supergroup includes an NDR region overlapping the TSS. Although a weak NDR was observed upstream of the first gene of pol II-transcribed PTUs in *T. brucei*, the pol II TSS remains unmapped. It is therefore unclear whether this NDR is functionally related to the transcription process or is due to a polypyrimidine tract that is required by the splicing mechanism, but is refractory to nucleosomes.

**Fig. 6:**
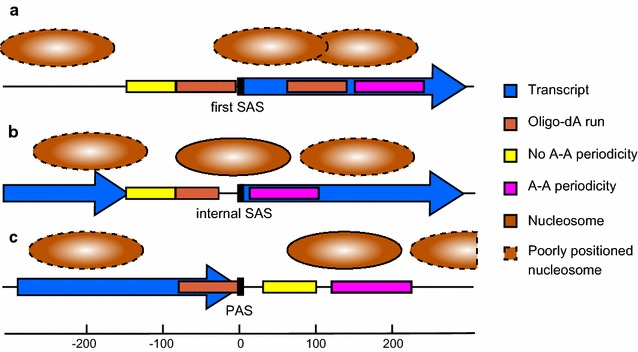
Model of nucleosomal organization at pol II-transcribed PTUs in *T. brucei*. Schematic representation of the average nucleosomal architecture and underlying DNA sequence positioning signals of *T. brucei* in a 400-bp window centered on (*a*) the first SAS of a PTU, representing the nucleosomal organization around the putative pol II TSS, (*b*) the internal SASs, and (*c*) the polyadenylation site

A detailed analysis of the DNA sequence in these regions showed that the NDR overlapped with a region of low distribution of A–A dinucleotides at a 10-bp periodicity, as well as with a region enriched for oligo-dA (see Fig. 6a). The distribution of A–A dinucleotides at a 10-bp periodicity is generally associated with anisotropically flexible DNA, amenable to tight spooling onto the histone octamers [63]. Oligo-dA, however, is flexurally rigid due to the presence of bifurcated hydrogen bonds and is thus not compatible with the bent path of nucleosomal DNA [64]. Oligo-dG is also averse to bending, due to the stacking of consecutive guanine bases [77]. It is unclear to what extent basal transcription factors and activators contribute to the observed NDR. Although the *T. brucei* genome does appear to encode transcription activators, none have yet been mapped to pol II PTU initiation regions. *T. brucei* does contain a TBP-related factor, TRF4. However, the protein motifs shown to interact with the DNA in *S.* *cerevisiae* TBP are absent in TRF4 [78]. TATA box elements have also not been identified in the *T. brucei* genome. Within the PTUs themselves, the internal SASs showed a single well-positioned nucleosome covering the SAS element. This nucleosome was similar to that seen at the 3′ end of a metazoan intron at the intron–exon boundary [54, 79], a splicing feature mechanistically related to a trypanosome SAS. This SAS nucleosome appears to incorporate the 5′ AG acceptor, where the nucleosome position may be directed by the oligo-dA run, present at a larger average distance upstream of the internal SAS compared to the first SAS (Fig. 6b).

In the case of the PASs, the oligo-dA abutting the end of the PAS would in theory be refractory for nucleosomes, whereas the presence of a weak A–A distribution at a 10-bp periodicity would accommodate the well-positioned nucleosome in its observed location (Fig. 6c). The nucleosomal organization seen at metazoan intron–exon boundaries and at polyadenylation sites, compared to that seen at *T. brucei* SASs and PASs, is thus highly similar. This could be a consequence of the evolutionarily conserved sequence elements that direct local nucleosome placement. Alternatively, this could be a nucleosome arrangement that is required for the respective genetic mechanisms involving these elements. The nucleosome organization at the SASs and PASs therefore appears evolutionarily ancient and was probably already established before the evolutionary divergence of the Excavata from the other eukaryotic supergroups [1, 80]. The aforementioned is supported by recent findings where comparable nucleosomal patterns were observed in *L. major*, a related kinetoplastid with genomic synteny to *T. brucei* [57]. The apparent organization of nucleosomes relative to these SAS and PAS sequence elements is intriguing, as these sequence elements, and notably the oligo-dA runs, are functionally relevant for RNA processing rather than transcription. Although the nucleosomal organization in the region of the first SAS is related to that seen at TSSs in other eukaryotes, this could be a consequence of the role of these sequences in RNA processing. It is possible that these sequences have dual functions in contributing both to the organization of the TSS region, as well as to spliceosome binding on the nascent RNA.

The functional relevance of the nucleosomal organization seen at internal SASs is less clear. One possibility is that the concerted placement of a nucleosome on internal SASs would slow the RNA polymerase at this position, ensuring time for splicing at the SAS and subsequent splicing and polyadenylation at the upstream PAS [81]. The juxtaposition of a poly-d(A·T) tract upstream of the nucleosome-associated internal SASs makes this an intriguing possibility, as the polypyrimidine tract is known to affect both *trans*-splicing and polyadenylation of adjacent genes [82]. It is also theoretically possible that factors involved in RNA processing are preloaded onto the DNA and then transferred to the growing, nascent RNA by the elongating pol II. In fact, a physical interaction between the SF3a60 spliceosome factor and the largest subunit of pol II was shown using a two-hybrid approach [83], suggesting a possible link between the elongating pol II and RNA splicing in *T. brucei*. In addition, TbRRM1, an RNA-binding SR nucleoprotein, has been shown to directly interact with histones and modulate chromatin structure, maintaining permissive chromatin to facilitate transcription and RNA processing, and may also be involved in splicing commitment for a subset of transcripts [84].

Alternatively, the nucleosomal presence at internal SASs (or the 3′ acceptor region of a metazoan intron) could limit DNA recombination in this area [85, 86]. This would protect exon units over evolutionary time, or limit mutation of the SAS, and thus conserve a mechanistically functional SAS. In support of this, the rate of C → T hydrolytic deamination was reported to be reduced by twofold in nucleosomally wrapped DNA in *S. cerevisiae*, *C. elegans*, and *Oryzias* [87]. Given that a single point mutation in the 3′ AG acceptor sequence can functionally destroy the entire downstream gene, it is likely that protection of such sites would be evolutionarily advantageous. The PAS sites themselves showed an NDR and a well-positioned nucleosome downstream, similar to the organization seen in other organisms [14]. This arrangement may also reflect a requirement for transient pausing of pol II transcription elongation.

Chromatin can play an important role in silencing areas of eukaryotic genomes. The regions flanking the silent *VSG* arrays showed a unique nucleosomal organization, with an extensive array of well-positioned nucleosomes. The nucleosomes on the silent *VSG* coding regions themselves appeared less well-positioned. We propose that the bordering chromatin structure of well-positioned nucleosomes serves to limit cryptic pol II initiation. For antigenic variation to work, it is imperative for *T. brucei* to maintain the silent *VSG* arrays in a transcriptionally repressed state. Promiscuous expression of these silent *VSGs* would allow the host immune system to develop an immune response to a wide variety of VSGs and therefore facilitate immune clearance of the parasite. This repressive nucleosomal arrangement found at the silent *VSG* arrays is reminiscent to that found at the silent mating-type loci *HML* and *HMR* in *S. cerevisiae*. In contrast, the less defined nucleosomal structure on the silent *VSG* genes themselves could be more permissive for gene conversion events copying the silent *VSG* to the active ES. In this way, the chromatin structure at these silent *VSG* arrays could facilitate antigenic variation in African trypanosomes.

In *T. brucei*, ORC1 and RAP1 were shown to be required for full repression of the silent ESs, and ORC1 was shown to bind at positions bordering many silent *VSG* cassettes. The similarity between silencing at the *S. cerevisiae* HM loci and telomeres, and silencing at the *T. brucei* ESs and the *VSG* gene arrays is striking. However, no Sir-related protein other than SIR2RP1 has been identified in *T. brucei*, and although SIR2RP1 is involved in silencing the immediate regions of the telomeres [29], it does not appear to play a direct role in ES silencing. In *S. cerevisiae*, *ORC1* is thought to be the ancestral gene of *SIR3*, which arose after a genome duplication event [88]. However, unlike yeast ORC1, the *T. brucei* ORC1 does not contain a BAH domain. It is therefore unclear whether it can substitute for SIR3 in *T. brucei* and therefore contribute to the propagation of a repressive heterochromatic structure at the telomeres and silent *VSG* arrays. Of all the mapped *T. brucei* ORC1 binding sites, 38% were found in the chromosomal cores, localizing to transcription boundaries, with the remaining 62% localizing to subtelomeric and silent *VSG* arrays. All active DNA origins of replication (ORI) were found in the chromosomal cores with no evidence of ORIs originating from subtelomeric sites or silent *VSG* arrays [74]. It is possible that these non-replicative ORC1 binding sites have a repressive function at transcriptionally silent *VSG*-containing subtelomeric regions in *T. brucei*. A gradient of repression extends up to 10 kb from the telomeric ends in *T. brucei* [30], implying the propagation of a repressive chromatin structure. However, the proteins that participate in the establishment of this repressive structure remain unknown.

## Conclusions

In summary, our genome-wide nucleosomal analysis revealed striking correlations, as well as stark differences, in the nucleosomal architecture of *T. brucei* compared to other model eukaryotes studied to date. These similarities suggest that some chromatin features, like the weak NDR upstream of PTUs, the strong positioning of nucleosomes on the internal SASs, the depletion of nucleosomes from PASs, and the nucleosomal organization at silent *VSG* arrays and ORC1 binding sites, were already established in LECA, before the divergence of the eukaryotic super groups. These findings, in conjunction with the co-localization of histone variants, histone and DNA modifications, and chromatin modulators, indicate the presence and importance of a functional epigenome in *T. brucei*, possibly providing a regulatory interface to genome regulation.

## Methods

### Trypanosome strains and culturing

Bloodstream form *T. brucei* Lister 427 was cultured in HMI-9 medium as previously described [89] supplemented with 15% fetal calf serum (FCS) and appropriate drugs at 37 °C under 5% CO_2_. Procyclic form trypanosomes were cultured in SDM-79 medium supplemented with 10% (v/v) FCS, 5 µg/ml hemin, and appropriate drugs at 27 °C [90]. Two cell lines were chosen for both the BF and the PF life cycle stages to account for possible cell line-specific differences. These are the BF (HNI_VO2 and RYT3) and PF (Amsterdam wild-type and 221BsrDsRed) cell lines. HNI_VO2 cells have a hygromycin resistance gene in the silent VSG221 ES and a neomycin resistance gene in the active VO2 ES, providing single copy sequences which allow the differentiation of the silent and active VSG ESs [91]. The RYT3 cell line has a blasticidin resistance gene in the active VSGT3 ES and an eGFP gene and a puromycin resistance gene in the silent VSG221 ES [32]. The PF 221BsrDsRed cell line has a blasticidin resistance gene and a DsRed gene in the silent 221 ES [32].

### Core particle preparation

MNase digestion of chromatin was performed as described [71]. Briefly, 5 × 10^7^ cells/sample were harvested by centrifugation (1200*g*, 5 min) and permeabilized with 400 µM digitonin for 5 min at room temperature (Sigma-Aldrich). Chromatin was digested with 1–32 units MNase (Worthington Biochemicals) for 5 min at 37 °C, followed by phenol–chloroform extraction and ethanol precipitation. Recovered DNA was resolved on a 2% (w/v) agarose gel. DNA fragments of ~147 bp were extracted from the gel using the freeze-and-squeeze technique (Bio-Rad laboratories, Hercules) and 50 nt from each end was sequenced by paired-end methodology (Illumina) as previously described [92].

### Alignment to reference genome

The FASTQ sequence files were aligned to version 4.2 of the *T. brucei* 427 genome using *Bowtie 2* [93] allowing no mismatches, limiting alignments to 7 ambiguous (N) bases in the reference genome, and filtering for convergent primer pairs separated by between 145 and 155 bp, end-to-end. The detail of the alignment output is shown in Additional file 1: Table S2.

### Dyad value files

The file of dyad values, listing the number of dyads assigned to each nucleotide position of each chromosome, was normalized to chromosome length to allow inter-chromosome comparisons, as well as normalized between different experiments, to allow analysis between different experimental conditions. We assumed that the median nucleosome density was the same between experimental conditions and chromosomes. The genomic positions of tandem repeats, previously identified with the *TRF* program [94], were downloaded from TriTrypDB, and the dyad values of all nucleotides that fell within tandem repeat sequences were set to “−1” with the program *strip_tandem_repeats*, and ignored in the calculation of average dyad densities. To mask the contribution of unmatched, N-rich sequences to the generation of spurious NDRs, all possible dyad positions of fragments between 145 and 155 bp, where either of the aligned 50-bp paired-end fragments exceeded the maximum cutoff for base ambiguity, were set to “−2” in the dyad value files, and ignored in subsequent calculations.

### Bin analysis

A binning analysis was performed with the program *dyad_bins* using the normalized dyad values. The lowest and highest numbers of dyad axes co-localized on single nucleotides, representing the minimum and maximum bin values, were identified. Dyad values associated with AT-rich repeats were masked as indicated. Bins intermediate to the minimum and maximum bins were defined at an increment of 1, and the number of occurrences of dyad values equal to each bin value identified in each file of dyad values. The number of members in each bin was normalized to the genome size to allow direct comparison between regions of different sizes. The number of samples in each bin was plotted with *Gnuplot* version 4.6.3.

### Normalized dyad densities

The number of dyads in a 10-bp scanning window was normalized with the program *dyads_genome_wide* to the number of nucleotides (excluding the “−1” and “−2” values), and expressed as the log_2_ of the ratio to the average number of dyads in the genome, as shown by the following equation:

$$\log_{2} \frac{{\frac{1}{N}\mathop \sum \nolimits_{i = 1}^{N} d_{i} }}{{\frac{1}{M}\mathop \sum \nolimits_{j = 1}^{M} d_{j} }}$$

where *N* is the width of the scanning window, *M* is the genome size, and *d*_*i*_ and *d*_*j*_ represent the number of assigned dyads at positions *i* and *j*, respectively.

### Differences in the nucleosomal organization in BF and PF *T. brucei*

The statistical significance of differences between the nucleosomal occupancy of each chromosome in the BF and PF life stage of *T. brucei* was determined with the Whitney–Mann *U* test. The autocorrelation function:

$$R_{k} = \frac{{\mathop \sum \nolimits_{i = 1}^{N - k} \left( {Y_{i} - \bar{Y}} \right)\left( {Y_{i + k} - \bar{Y}} \right)}}{{\mathop \sum \nolimits_{i = 1}^{N} \left( {Y_{i} - \bar{Y}} \right)^{2} }}$$

where *N* is the number of data points, *k* the offset, *Y* the average value for the dataset, and *Y*_*i*_ and Y_*i*+*k*_ the values at positions *i* and *i* + *k*, and it was applied to the log_2_ dyad values and smoothed with a 10-bp running average. The autocorrelation plot showed a significant flattening of the autocorrelation coefficient at values beyond *k* = 10 (Additional file 1: Fig. S10), as expected. We therefore chose values 50 nt apart to ensure independence of data points, as is required by the statistical test. The nonparametric Whitney–Mann *U* test was performed in a scanning 500-bp window at consecutive settings on each chromosome, using data points at 50-bp intervals. Statistical significance was determined by using a Whitney–Mann *U* value *p* < 0.01, and the presence of a 500-bp setting that was statistically significant between the BF and PF stages, recorded at the start of each window setting. To increase the rigor of the significance test, we further summed the number of statistically significant settings in a 10-bp window for the two biological replicates as well as the two different *T. brucei* cell lines, to correct for possible cell line related differences. Only cumulative statistical significance peaks in the top 10th percentile of the range were tagged for further investigation.

### Alignment of dyads

The alignment of dyads relative to specific genomic positions was performed with the program *align_dyads*, which aligned the dyads in an orientation depending on whether the feature was on the Watson or the Crick strand, and reported the average value in a 50-bp running window.

### Statistical test

The Kendall’s tau correlation and Pearson product moment correlation test were performed with R scripts.

### Mapping of *T. brucei* 927 genes to *T. brucei* 427

The list of translated coding sequences of *T. brucei* strain 927 (Tb927) was downloaded from TriTypDB and BLASTed against a database of *T. brucei* 427 protein sequences (Tb427). This had been generated with version 7 of the fastA format translated CDS files downloaded from tritrypdb.org, using the *makeblastdb*. The homologous sequences were listed, and entries with *E* values <10^−10^ selected. Where Tb927 genes were mapped to multiple Tb427 genes, only the match with the smallest *E* value was selected. Where a Tb927 gene had more than one splice acceptor site, the site furthest upstream from the coding sequence was chosen as it was likely to be closest to the site of transcription initiation. The position of the splice acceptor sites in the genome sequence of the Tb427 was mapped using the program *927_to_427_map*.

### Mapping of ORC1 binding sites from Tb927 to Tb427

The ORC1 binding sites assigned with a signal ratio >1.0 and a FDR <0.05 were selected from the list of ORC1 sites (kindly provided by R. McCulloch) [74], and the corresponding sequences in Tb927 retrieved from TriTrypDB. The retrieved sequences were BLASTed against version 4.2 of the *T. brucei* Lister 427 genome, and the single, best hit for each query sequence recovered. Only target sequences that were >95% identical and with *E* values <10^−10^ were retained (1114 sites, Additional file 2: Table S3). The ORC1 site was defined as the center of each such recovered sequence.

### Definition of chromosome regions

Subtelomeric regions were identified as the terminal regions of chromosomes enriched for retrotransposon hotspot proteins (*RHS*), expression site-associated genes (*ESAGs*), *VSG* genes and pseudogenes, and leucine-rich repeat protein (*LRRP*) genes. Subtelomeric regions were further divided into telomere–proximal (≤10% of the chromosomal length from the telomere) and chromosome internal/core (>10% of the chromosomal length away from telomere) subtelomeric regions.

### Alignment to resistance markers

The hygromycin resistance gene (Hygromycin-B 4-O-kinase; accession number V01499) as well as the neomycin resistance marker (aminoglycoside 3′-phosphotransferase; accession number P00551) was indexed using Bowtie 2 with default settings, and the sequenced pairs for the BF HNI_VO2 aligned to the indexed sequences, allowing accordant alignments of sequence pairs resulting in fragment lengths of between 145 and 155 bp.

### Probability of sequence repeats

Nucleotide repeat sequences were identified in the *T. brucei* genome with *polyA_genome_distribution*. The occurrence of runs between specific genomic positions, such as within transcription start regions, was identified with the program *in_or_out.* The probability of specific sequence repeats occurring in the genome was calculated as a hypergeometric distribution. Where the sample size *n* ≪ *N*, the population size, the hypergeometric distribution can be approximated as:

$$\frac{1}{{f^{n} }}$$

where *n* is the oligonucleotide sequence length and *f* is the fractional occurrence of the given nucleotide. The fractional occurrence of A·T and G·C in the genome is 0.2651 and 0.2349, respectively. The probability of occurrence of each oligonucleotide decamer with a given dinucleotide composition was calculated and is shown in Additional file 1: Table S1.

### Probability of finding an A10, G10, and AT10 repeat

Specific sequences were recovered from the genome of *T. brucei* using the program *get_sequences*. The enrichment of specific nucleotide oligomers was determined in aligned sequences using the program *polyA_enrich*. The number of sequence runs was normalized to the number of sequences analyzed, smoothed with a 50-nt running average, and expressed as a percentage of the smoothing window size. The probability of finding 10-bp homo- or hetero-oligomer motifs in the coding and noncoding regions of *T. brucei* 427 was calculated with a hypergeometric distribution function using the coding frequencies shown in Additional file 1: Table S4.

The transcription start regions identified in Tb927 were mapped to the equivalent genomic positions in Tb427 using the correlation list derived from the BLAST analysis explained above. The presence of upstream *t*-, *r*- *or snRNA* genes was verified for internal start regions. The list of mapped transcription start regions is shown in Additional file 3: Table S5.

### Discrete Fourier analysis of dinucleotide frequencies

The presence of strong 10-bp A–A dinucleotide periodicities was assessed from the Fourier magnitude of the distribution in specific regions using the program *hp_fftw* that utilizes the *FFTW* library (www.fftw.org).

### Software

All software was written in C++ (ISO/IEC 14882:2011) and compiled with the g++ version 4.8.2 64-bit compiler (gcc.gnu.org) using the mingw-w64 version 4.8.2 (mingw-w64.sourceforge.net) toolchain on a Windows version 8.1 operating system platform. The program *hp_fftw* was compiled with g++ version 4.8 on Linux openSUSE version 12.3 (www.opensuse.org). All plots were prepared with scripts using gnuplot version 4.6 (www.gnuplot.info). The source code for software developed and used in this study is freely available (sourceforge.net/projects/nucpos/).

## Additional files

**Additional file 1.** Supplementary nucleosome dyad profiles, oligonucleotide runs, (di)nucleotide occurrence and frequencies, megabase chromosome panels, and auto-correlation of nucleosome positions

**Additional file 2: Table S3.** Genomic positions of equivalent sequences mapped as ORC1 sites in *T. brucei* strain 927 in *T. brucei* strain 427.

**Additional file 3: Table S5.** Mapped transcription start regions from Tb927 to Tb427.

## Abbreviations

LECA: last eukaryotic common ancestor
pol II: RNA polymerase II
TSS: transcription start site
HAT: human African trypanosomiasis
BF: bloodstream form
PF: procyclic form
VSG: variant surface glycoprotein
ES: expression site
PTU: polycistronic transcription unit
SSR: strand switch region
Base J: β-d-glucosyl-hydroxymethyluracil
MNase: micrococcal nuclease
NDR: nucleosome-depleted region
SAS: splice acceptor site
PAS: polyadenylation site
SL RNA: spliced leader RNA
PPT: polypyrimidine tract
RHS: retrotransposon hotspot protein
ESAG: expression site-associated gene
LRRP: leucine-rich repeat protein
FDR: false discovery rate
ORI: origins of replication
FCS: fetal calf serum
