# Supplementary material for: Well-positioned nucleosomes punctuate polycistronic pol II transcription units and flank silent VSG gene arrays in Trypanosoma brucei
Source: Epigenetics Chromatin. 2017 Mar 20;10:14. doi: 10.1186/s13072-017-0121-9 (PMC5359979; doi:10.1186/s13072-017-0121-9)
Supplement: Supplementary file 1 — Additional file 1. Supplementary nucleosome dyad profiles, oligonucleotide runs, (di)nucleotide occurrence and frequencies, megabase chromosome panels, and auto-correlation of nucleosome positions [file 13072_2017_121_MOESM1_ESM.pdf]

Additional File 1: Supplementary Information

Maree et al.

Well positioned nucleosomes punctuate polycistronic Pol II transcription units and flank silent *VSG* gene arrays in *Trypanosoma brucei*.

Johannes Petrus Maree[1ǂ], Megan Lindsay Povelones[2], David Johannes Clark[3], Gloria Rudenko[4], Hugh–George Patterton[1*ǂ]

1. Department of Biochemistry, Stellenbosch University, Matieland 7602, South Africa.

2. Department of Biology, Pennsylvania State University (Brandywine Campus), Media, Pennsylvania 19063, United States of America

3. Division of Developmental Biology, Eunice Kennedy Shriver National Institute for Child Health and Human Development, National Institutes of Health, Bethesda, MD, United States of America

4. Department of Life Sciences, Imperial College London, South Kensington, London SW7 2AZ, United Kingdom

*Corresponding Author: Tel: +27-21-8082774; Email: hpatterton@sun.ac.za

ǂ As members of the H3Africa Consortium

Keywords: genome-wide nucleosome positions, *Trypanosoma brucei*, polycistronic transcription units, silent variant surface glycoproteins, bloodstream expression sites, evolution, MNase-seq

Supplementary Figures

A

```
                                                                                    A box
S. cerevisiae           GGTTGCGGCCATATCTACCAGAAAGCACCGTTTCCCGTCCGATCAACTGTAGTTAAGCTGG
Xenopus                 GCCTACGGCCACACCACCCTGAAAGTGCCTGATCTCGTCTGATCTCAGAAGCGATACAGGG
T. brucei               GGGTACGACCATACTTGGCCGAATGCACCATATCCCGTCCGATTTGTGAAGTTAAGCGGCC
                        *  * ** *** *     * *** *  **   ** **** ***

                                  I             C box
S. cerevisiae           TAAGAGCCTGACCGAGTAGTGTAGTGGGTGACCATACGCGAAACTCAGGTGCTGCAATCT
Xenopus                 -TCGGGCCT-GGTTAGTACCTGGATGGGAGACCGCCTGGGAATACCAGGTGTCGTAGGCT
T. brucei               -ACAGGCCT-CGTTAGTACGGCGATCAGTGATGGCGCTGGAACCCGGGGTGTTGTACTCT
                             ****     ****      *  * **        ***     ****  * *  **
```

B

```
           10        20         30
-         .-CAUACU      AAU-| AC    UCCCG
 GGGUACGAC        UGGCCG    GC  CAUA     U
 CUCAUGUUG        ACCGGC    UG  GUGU     C
U         \ ------      GAAU^ AA    UUAGC
       110         60        50        40

                  70        80
            CAG      UUAGUA     A   G
               GCCUCG      CGGCG UCA U
               UGGGGC      GUCGC GGU G
            ---      CCAAG-     -   A
                      100          90
```

Figure S1. The internal control regions of the *T. brucei 5S rRNA* gene. A) Multiple alignment of the *S. cerevisiae*, *Xenopus* and *T. brucei 5S rRNA* genes. The locations of the A box, intermediate element (I) and C box, identified in the *S. cerevisiae* gene, are indicated. The A and C boxes localize to the B and D loops of the folded *5S rRNA*, respectively. **B**) Predicted folding of the *T. brucei* 5S rRNA transcript using Mfold (http://mfold.rna.albany.edu/). The positions of the *T. brucei* sequences that aligned with the *S. cerevisiae* A and C boxes in panel **A**, are shown, localizes to the predicted B and D loops.

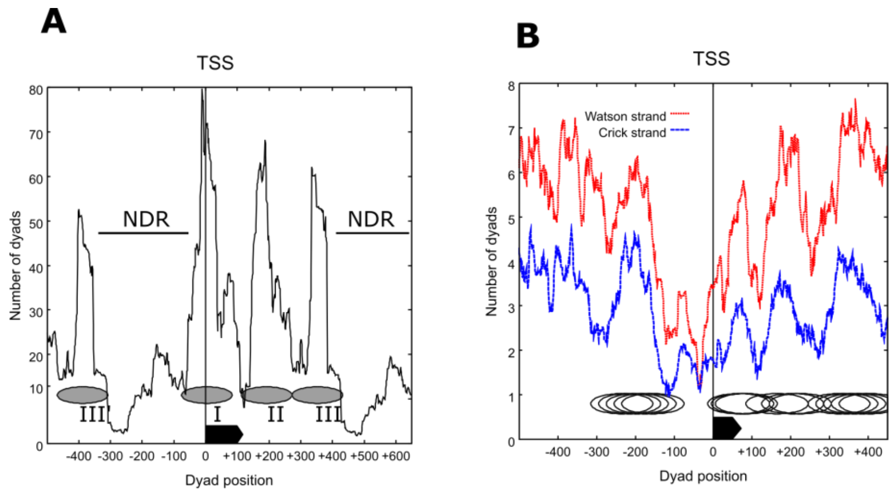

Figure S2. Alignment of dyad axes relative to pol III transcribed loci in PF cells. Genes were aligned relative to the transcript start (tritrypdb.org), indicated by the vertical black line at dyad position 0 in each panel. **A**) The average, cumulative dyad distribution is shown for *5S rRNA* genes. **B**) Separate alignment of Watson and Crick strand data relative to the transcription start site of *tRNA* genes in BF cells

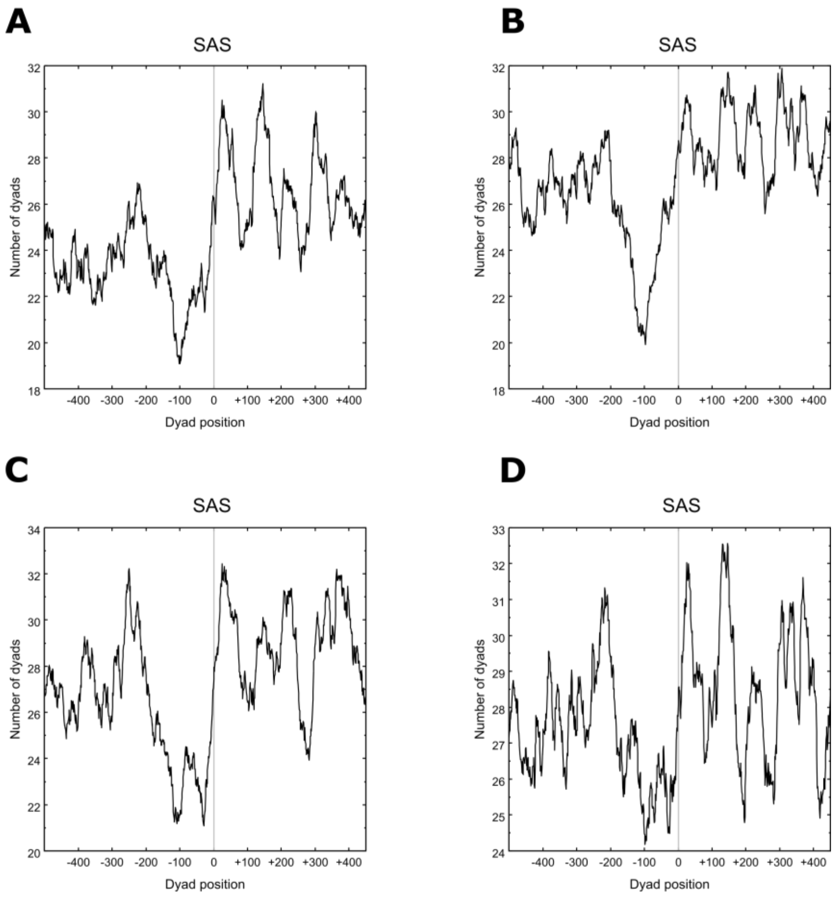

Figure S3. Alignment of dyad axes in biological duplicates from two different BF *T. brucei* cell lines. The deduced positions of nucleosome dyad axes were aligned relative to the most distal SAS for the first gene of every PTU. **A**) BF *T. brucei* HN1_VO2, sample 1. **B**) BF *T. brucei* HN1_VO2, sample 2. **C**) BF *T. brucei* RYT3, sample 1. **D**) BF *T. brucei* RYT3, sample 2.

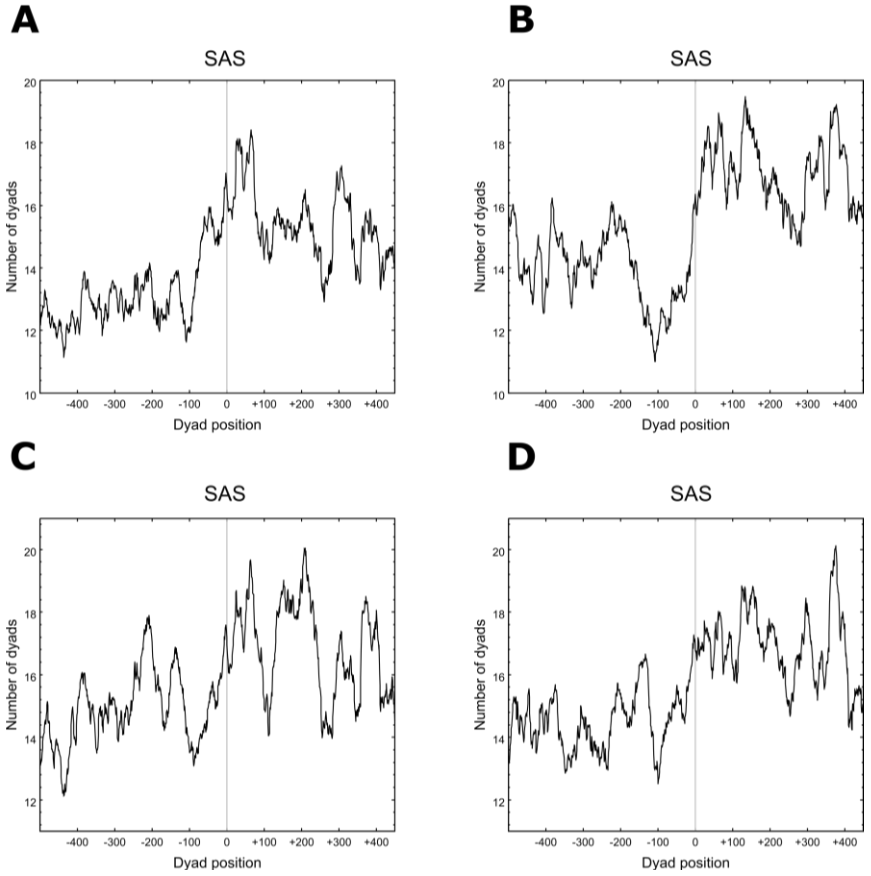

Figure S4. **Alignment of dyad axes in biological duplicates from two different PF *T. brucei* cell lines.** The deduced positions of nucleosome dyad axes were aligned relative to the most distal SAS for the first gene of every PTU. **A**) *T. brucei* Amsterdam WT, sample 1. **B**) *T. brucei* Amsterdam WT, sample 2. **C**) *T. brucei* DsRed, sample 1. **D**) PF *T. brucei* DsRed, sample 2.

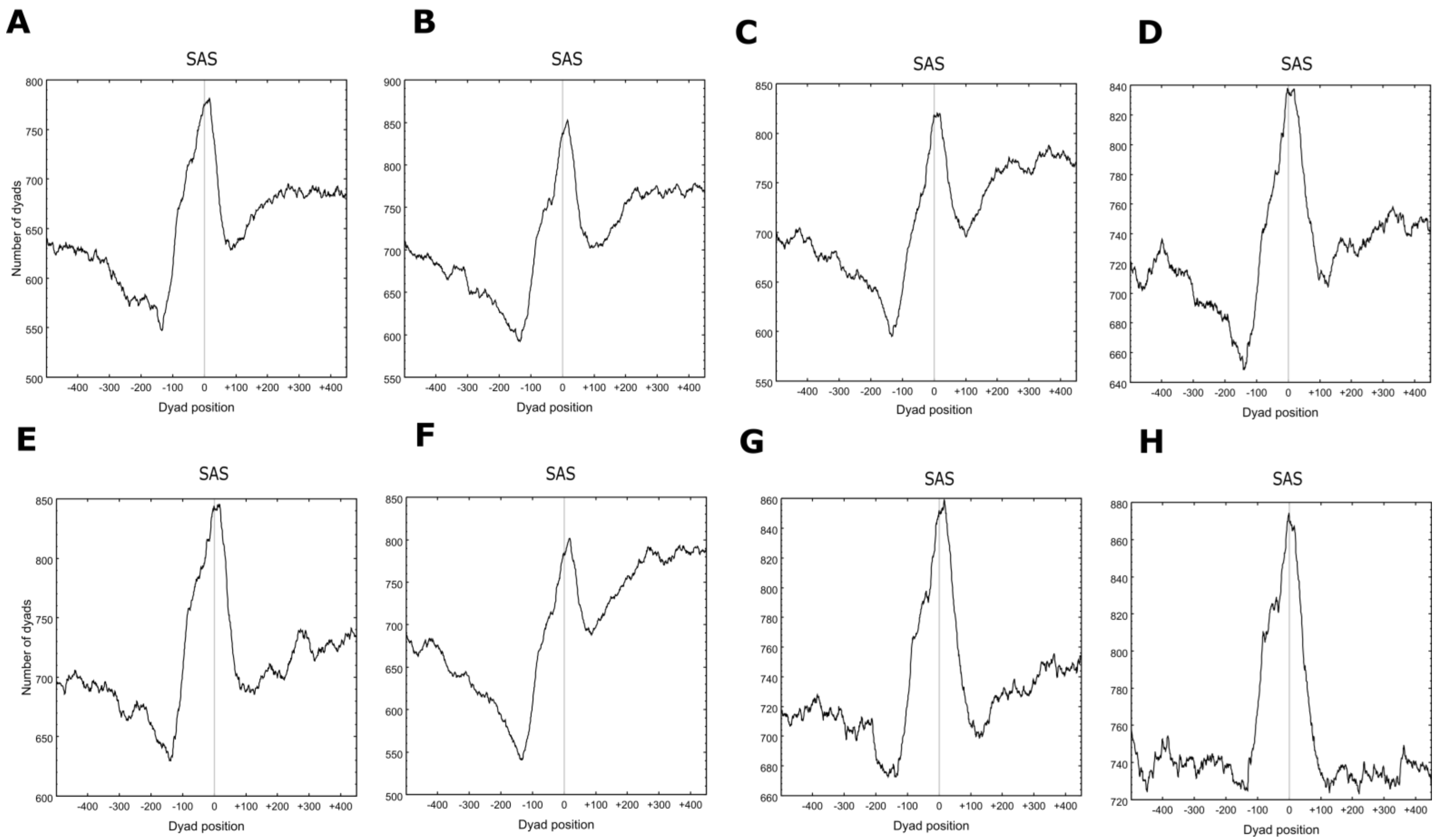

Figure S5. Nucleosomal dyad axes in BF and PF *T. brucei* cell lines aligned relative to the SAS of all genes in all PTUs. The deduced positions of the nucleosomal dyad axes of four BF and four PF *T. brucei* samples were aligned relative to the SAS for all mapped genes in all PTUs. **A**) BF *T. brucei* HN1_VO2, sample 1. **B**) BF *T. brucei* HN1_VO2, sample 2. **C**) BF *T. brucei* RYT3, sample 1. **D**) BF *T. brucei* RYT3, sample 2. **E**) PF *T. brucei* Amsterdam WT, sample 1. **F**) PF *T. brucei* Amsterdam WT, sample 2. **G**) PF *T. brucei* DsRed, sample 1. **H**) PF *T. brucei* DsRed, sample 2.

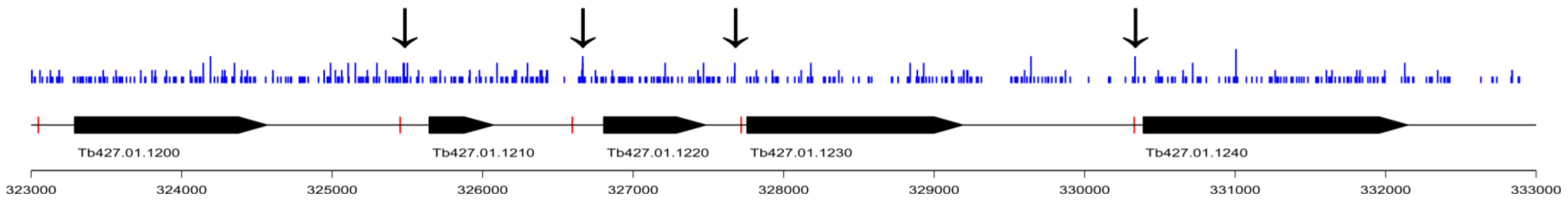

Figure S6. Distribution of nucleosomal dyads at the SAS of individual genes. The normalized distribution of nucleosomal dyad axes is shown for a region of chromosome 1 (323000 – 333000 showing genes Tb427.01.1200 to Tb427.01.1240). The position of the most distal SAS for each of the indicated genes are shown by the vertical red bar. The sequence positions are indicated at the bottom of the figure. Co-aligned dyads, indicative of positioned nucleosomes, are indicated by the arrows.

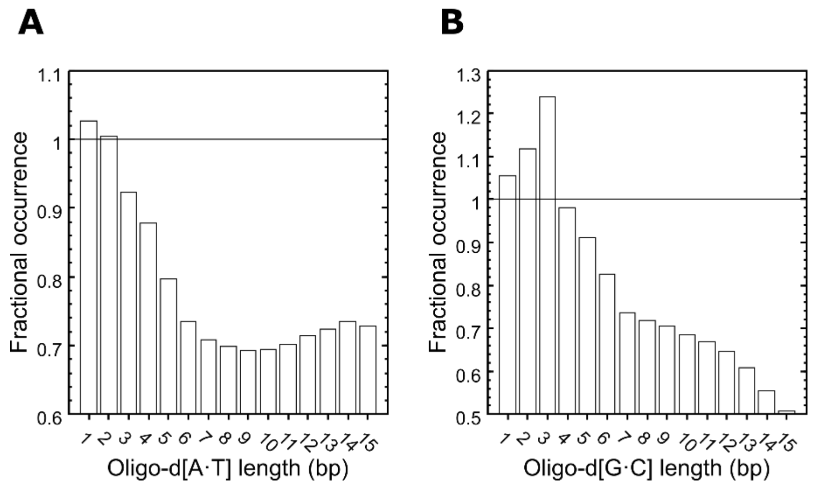

Figure S7. Presence of oligo-d[A·T] and oligo d[G·C] in nucleosomal DNA. The average occurrence of **A**) oligo-d[A·T] and **B**) oligo d[G·C] runs of 1-15 bp in 147 bp regions centered on assigned dyads are shown normalized to the average occurrence of the same sequence in all possible nucleosome settings in the *T. brucei* genome.

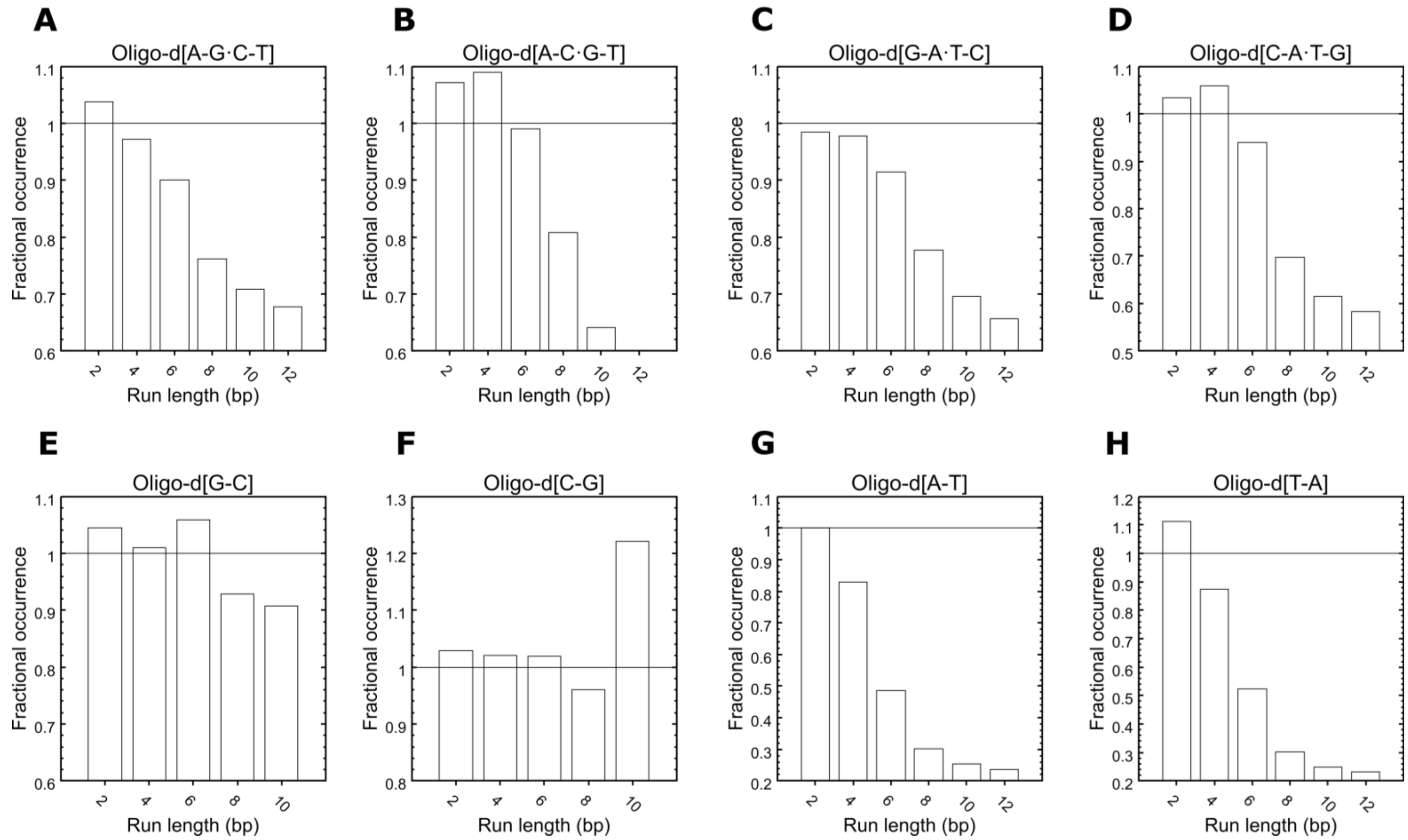

Figure S8. Presence of dinucleotides in nucleosomal DNA. The average occurrence of **A**) oligo-d[A-G·C-T], **B**) oligo-d[A-C·G-T], **C**) oligo-d[G-A·T-C], **D**) oligo-d[C-A·T-G], **E**) oligo-d[G-C], **F**) oligo-d[C-G], **G**) oligo-d[A-T] and **H**) oligo-d[T-A] runs of 2-12 bp in 147 bp regions centered on assigned nucleosomal dyads is shown normalized to the average occurrence of the same sequence in all possible settings of the corresponding size in the *T. brucei* genome. Note that runs of oligo-d[G-C] and oligo-d[C-G] of 12 bp and longer do not occur in the *T. brucei* genome.

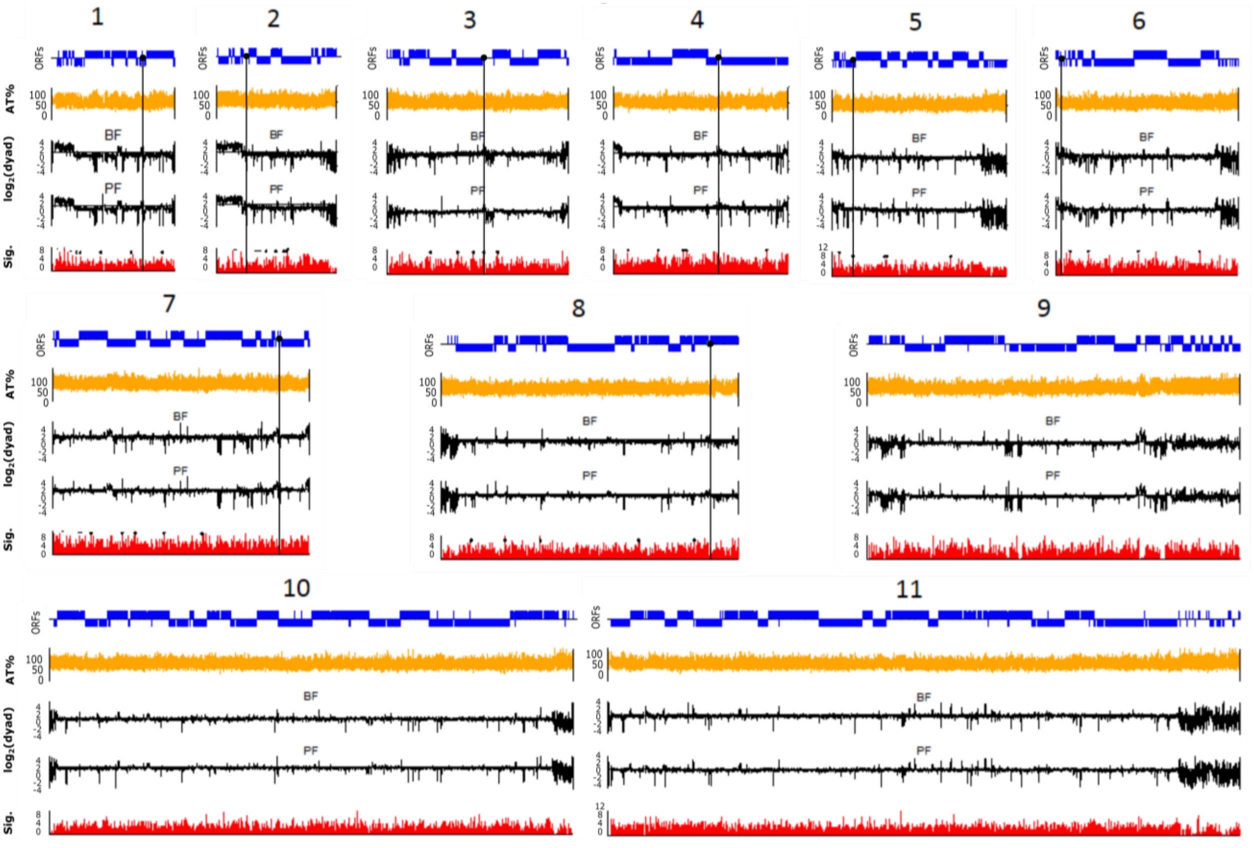

Figure S9. Nucleosomal organization of the megabase chromosomes in BF and PF *T. brucei*. Each of the megabase chromosomes are shown in panels 1 to 11, with the top line indicating the position of assigned genes on the Watson strand (blue lines above the horizontal axis) and Crick strand (blue lines below the horizontal axis). The locations of the centromeres are indicated by the filled circles and black vertical lines for chromosomes 1-8. The next line shows the percentage of A/T nucleotides at each setting of a 100 bp scanning window. This is followed by a trace of the $\log_2$ ratio of the average number of assigned dyad axes in a 1000 bp scanning window to the genome average in BF and PF *T. brucei*. The correlation between the relative nucleosome density in BF and PF cultures was calculated by the Whitney-Mann U test at 50 bp intervals in a 500 bp sliding window. The number of statistically significant ($p<0.01$) samples in four biological replicates were added, and is shown as a significance value (Sig.) by the red line in the bottom trace. The small black circles identify peaks of high significance where the nucleosomal organization of the corresponding regions were individually assessed for chromosomes 1 to 8.

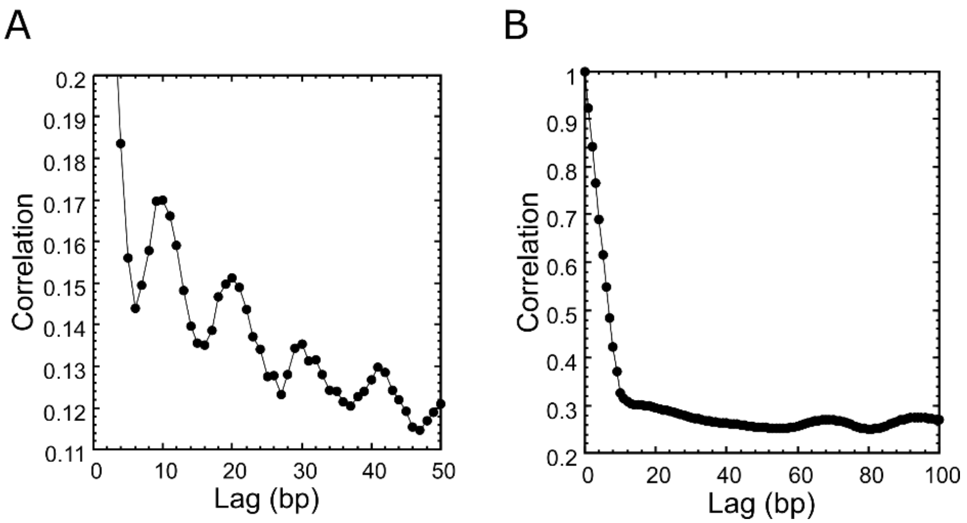

Figure S10. Auto-correlation of nucleosome positions. An auto-correlation analysis was performed to assess interdependence of data points, or how data points influence each other, in order to choose values (nucleotides) that were far enough apart to ensure independence of data points, as required for the Whitney-Mann Wilcoxon U test. This shows in **A**) the log2 of the ratio of the average dyad density in a scanning window to that of the genome average, and **B**) the normalized number of dyads assigned to each nucleotide position.

Supplementary Tables

Table S1. Representation of different 10-mer oligonucleotides in the genome of *T. brucei* 427.

Monomer unit[1]	Expectancy[2]	Number in genome[3]	Occurrence/10 bp setting[4]	Fold representation[5]	Fractional occurrence[6]
GG	5.12×10^{-7}	858	3.35×10^{-5}	65	0.26
GA	9.36×10^{-7}	792	3.09×10^{-5}	33	0.13
GT	9.36×10^{-7}	4428	1.73×10^{-4}	185	0.74
GC	5.12×10^{-7}	22	8.59×10^{-7}	2	0.01
AG	9.36×10^{-7}	846	3.30×10^{-5}	35	0.14
AA	1.71×10^{-6}	9854	3.85×10^{-4}	225	0.90
AT	1.71×10^{-6}	10955	4.28×10^{-4}	250	1.00
AC	9.36×10^{-7}	4240	1.66×10^{-4}	177	0.71
TG	9.36×10^{-7}	4572	1.79×10^{-4}	191	0.76
TA	1.71×10^{-6}	10156	3.97×10^{-4}	232	0.93
TT	1.71×10^{-6}	9390	3.67×10^{-4}	214	0.86
TC	9.36×10^{-7}	886	3.46×10^{-5}	37	0.15
CG	5.12×10^{-7}	14	5.47×10^{-7}	1	0.004
CA	9.36×10^{-7}	4343	1.70×10^{-4}	181	0.72
CT	9.36×10^{-7}	943	3.68×10^{-5}	39	0.16
CC	5.12×10^{-7}	978	3.82×10^{-5}	75	0.30

1. The repeating dinucleotide unit in the 10 bp oligonucleotide run.
2. Calculated as $1/f^n$ where f is the genomic frequency of a nucleotide, and n is the number of nucleotides in the run. The genomic frequency of nucleotides in version 4 of the *T. brucei* Lister 427 genome is G/C = 0.2349 and A/T = 0.2651.
3. The number of times the 10 bp oligonucleotide occurs the *T. brucei* genome.
4. The number of occurrences as a fraction of the total number of 10 bp settings in the genome
5. Fold difference between the observed occurrence per 10 bp setting, and the theoretical predicted frequency
6. The fractional occurrence relative to the most abundant oligomer

Table S2. Detail of alignment output. Detail of alignment of paired-end sequences to version 4 of the *Trypanosoma brucei* 427 genome (www.tritrypdb.org) using Bowtie version 2 (bowtie-bio.sourceforge.net).

Sample	No match	Concordant alignments (1 match)	Concordant alignments (>1 match)	Discordant alignments	Total reads	Fold Coverage	Alignment rate (%)
1. BF HNI_VO2	4242377	5423632	2163826	98774	11829835	23	68
2. BF HNI_VO2	4794897	6459546	2573654	91225	13828097	27	69
3. PF Amt WT	3960221	5606947	2394925	37317	11962093	23	70
4. PF Amt WT	3857769	6074342	2587847	43107	12519958	24	72
5. BF RYT3	3828404	6469307	2581145	51115	12878856	25	73
6. BF RYT3	4389075	6786489	2668326	43988	13843890	27	71
7. PF 221BsrDsRed	4199013	6464713	2809759	24169	13473485	26	71
8. PF 221BsrDsRed	4415091	5796994	2532050	118453	12744135	25	70

Table S3. Genomic positions of equivalent sequences mapped as ORC1 sites in *T. brucei* 927 in *T. brucei* 427. Included as additional file 2.

Table S4. Nucleotide occurrence and frequencies in assigned genes and intergenic regions of the *Trypanosoma brucei* 427 version 4 genome (tritrypdb.org).

Ambiguous bases in the sequence were ignored.

		Genome	Coding	Intergenic[b]
Number	A	6,508,018	2,977,079	3,530,939
	T	6,510,467	2,993,300	3,517,167
	G	5,747,024	3,074,257	2,672,767
	C	5,789,917	3,083,330	2,706,587
	Sum	24,555,426[a]	12,127,966	12,427,460
Percentage	A%	26.50338	24.54722	28.41239
	T%	26.51335	24.68097	28.30158
	G%	23.40429	25.3485	21.50695
	C%	23.57897	25.42331	21.77908

[a]The genome size excludes ambiguous bases (N)
[b]Includes telomeric sequences

Table S5. Mapped transcription start regions from Tb927 to Tb427. Included as additional file 3
